# The SOS response-induced cell division inhibitor YneA interacts with FtsW to block septal peptidoglycan synthesis in *Bacillus subtilis*

**DOI:** 10.1016/j.jbc.2026.113276

**Published:** 2026-06-23

**Authors:** Wenjie Yang, Chao Wang, Yuanyuan Cui, Wanting Jiang, Shimin Zhu, Changjiang Dong, Joe Lutkenhaus, Xiangdong Chen, Shishen Du

**Affiliations:** 1 State Key Laboratory of Metabolism and Regulation in Complex Organisms, College of Life Sciences, Wuhan University, Wuhan, Hubei, China; 2 Hubei Key Laboratory of Cell Homeostasis, College of Life Sciences, Wuhan University, Wuhan, Hubei, China; 3 State Key Laboratory of Virology and Biosafety, College of Life Sciences, Wuhan University, Wuhan, Hubei, China; 4 Key Laboratory of Combinatorial Biosynthesis and Drug Discovery, Ministry of Education, School of Pharmaceutical Sciences, Wuhan University, Wuhan, China; 5 Department of Microbiology, Molecular Genetics and Immunology, University of Kansas Medical Center, Kansas City, Kansas, USA

**Keywords:** SOS response, cell division, septal peptidoglycan synthesis, FtsW, YneA, peptidoglycan polymerase

## Abstract

Maintenance of genome integrity is critical for the survival and proliferation of bacteria. Following DNA damage, many bacteria launch the SOS response, which up-regulates many enzymes involved in DNA damage repair, as well as cell division inhibitors which coordinate DNA repair and cell division. YneA is a potent SOS response-inducible cell division inhibitor in *Bacillus subtilis*. It consists of a single transmembrane (TM) domain linked to an extracellular LysM domain. Previous studies suggest that YneA interferes with divisome assembly and septal peptidoglycan (sPG) synthesis, however, the underlying mechanism remains unclear. In this study, we show that overexpression of YneA does not disrupt divisome assembly but blocks sPG synthesis. It localizes to the division site through an interaction between its TM domain and TM7 of FtsW, which is a SEDS-family peptidoglycan polymerase that partners with PBP2B (FtsI) to synthesize sPG. Mutations in the TM domain of YneA and the TM7 of FtsW eliminate YneA localization to midcell and its toxicity. Moreover, its ability to bind PG through its LysM domain is essential for inhibition of sPG synthesis. Taken together, these results indicate that YneA inhibits cell division by targeting FtsW and blocking its PG synthetic activity. Intriguingly, AlphaFold 3 structure predictions suggest that several SOS response-inducible cell division inhibitors also target FtsW, implying that inhibition of FtsW function is a common way to block cell division during SOS response.

Coordination of DNA replication and cell division is critical for the survival and reproduction of bacteria (1, 2, 3). To prevent the passage of damaged genetic material to daughter cells, bacteria have evolved various mechanisms to delay cell division upon DNA damage, allowing time for repair of damaged DNA. A nearly universal response to DNA damage in bacteria is the LexA-mediated SOS response, which is initiated by the binding of the RecA protein to single-stranded DNA generated during DNA damage (3, 4, 5, 6, 7, 8, 9, 10, 11). Formation of RecA-ssDNA filaments leads to the autocleavage of the master transcriptional repressor LexA (12, 13, 14, 15, 16, 17, 18), resulting in the activation of the SOS response. Subsequently, many genes involved in DNA repair and coping with this stress are up-regulated, including cell division inhibitors (3, 11, 19, 20).

The best studied SOS response-inducible cell division inhibitor is SulA in *Escherichia coli*, which interacts directly with the highly conserved cell division protein FtsZ (21, 22, 23, 24, 25, 26, 27). FtsZ is a tubulin-like GTPase, which polymerizes into the Z-ring at midcell to initiate cell division in nearly all bacteria (28, 29). Z-ring functions as a scaffold for the assembly of the divisome which synthesizes septal peptidoglycan (sPG), leading to the constriction of the cells (30, 31). SulA inhibits cell division by preventing FtsZ polymerization (25, 32, 33, 34, 35). Following the repair of damaged DNA, SulA synthesis is prevented and it is rapidly degraded by the protease Lon, enabling the cell to resume division (23, 36). Although SulA-mediated inhibition of Z-ring formation appears to be a highly effective way to block cell division upon DNA damage, it is not broadly conserved among bacteria (3). Recent studies found that several bacteria employ small integral membrane proteins to coordinate cell division with DNA damage repair (3, 37, 38, 39, 40, 41, 42). In *Caulobacter crescentus,* the DNA damage induced cell division inhibitors SidA and DidA do not interfere with Z ring formation or divisome assembly, but interact directly with the components of the sPG synthetic complex, FtsW or FtsN (40, 41). Likewise, in *Mycobacterium tuberculosis* and *Staphylococcus aureus,* the DNA damage-induced division inhibitors ChiZ and SosA, respectively, do not affect the localization of FtsZ at midcell but target late steps of division (38, 42, 43, 44). It seems that utilization of small integral membrane proteins to block a late step in cell division is a widespread mechanism during the SOS response.

In *Bacillus subtilis*, a small bitopic membrane protein YneA is induced by the SOS response to inhibit cell division (37). It is counteracted by its antagonist DdcA and is quickly degraded by two membrane-bound proteases, CtpA and DdcP (45, 46). As a consequence, cells can rapidly resume division after the damaged DNA has been repaired (37, 45, 46, 47, 48). YneA contains an N-terminal transmembrane (TM) domain and a C-terminal LysM domain which is a canonical PG binding motif (47, 49, 50). The mechanism by which YneA inhibits cell division has been extensively studied. Mutations in the TM and LysM domains of YneA reduce or abolish its cell division inhibitory activity, indicating that both domains are necessary for its function (47, 48). Moreover, YneA has been shown to interact with several division proteins, including FtsL, PBP2B (FtsI) and PBP1, in Bacterial Two Hybrid (BTH) assays (48). However, mutations in FtsW, a SEDS-family peptidoglycan polymerase dedicated for sPG synthesis, but not any of the above mentioned proteins, are refractory to the cell division inhibitory activity of YneA (48). Analysis of one such FtsW mutation, L196P (mis-numbered as L148P in the report), showed that it significantly reduced cell filamentation caused by YneA upon DNA damage (48). It was assumed that FtsW^L196P^ hyperactivated the divisome such that it conferred resistance to YneA, even though it did not reduce cell length in the absence of DNA damage (shorter cell length is typical for activation mutants). Based on these results, it has been proposed that YneA interferes with interactions among late-arriving proteins FtsL/PBP2B/PBP1 and thereby blocking sPG synthesis (47, 48). However, it remains to be determined which divisome protein is the primary target of YneA and how it blocks sPG synthesis.

In this study, we discovered that YneA does not prevent divisome assembly but interacts with FtsW through its TM domain to localize to the divisome and then likely binds newly synthesized sPG *via* its LysM domain to block sPG synthesis.

## Results

### YneA blocks septal peptidoglycan synthesis but not divisome assembly

**Figure 1:**
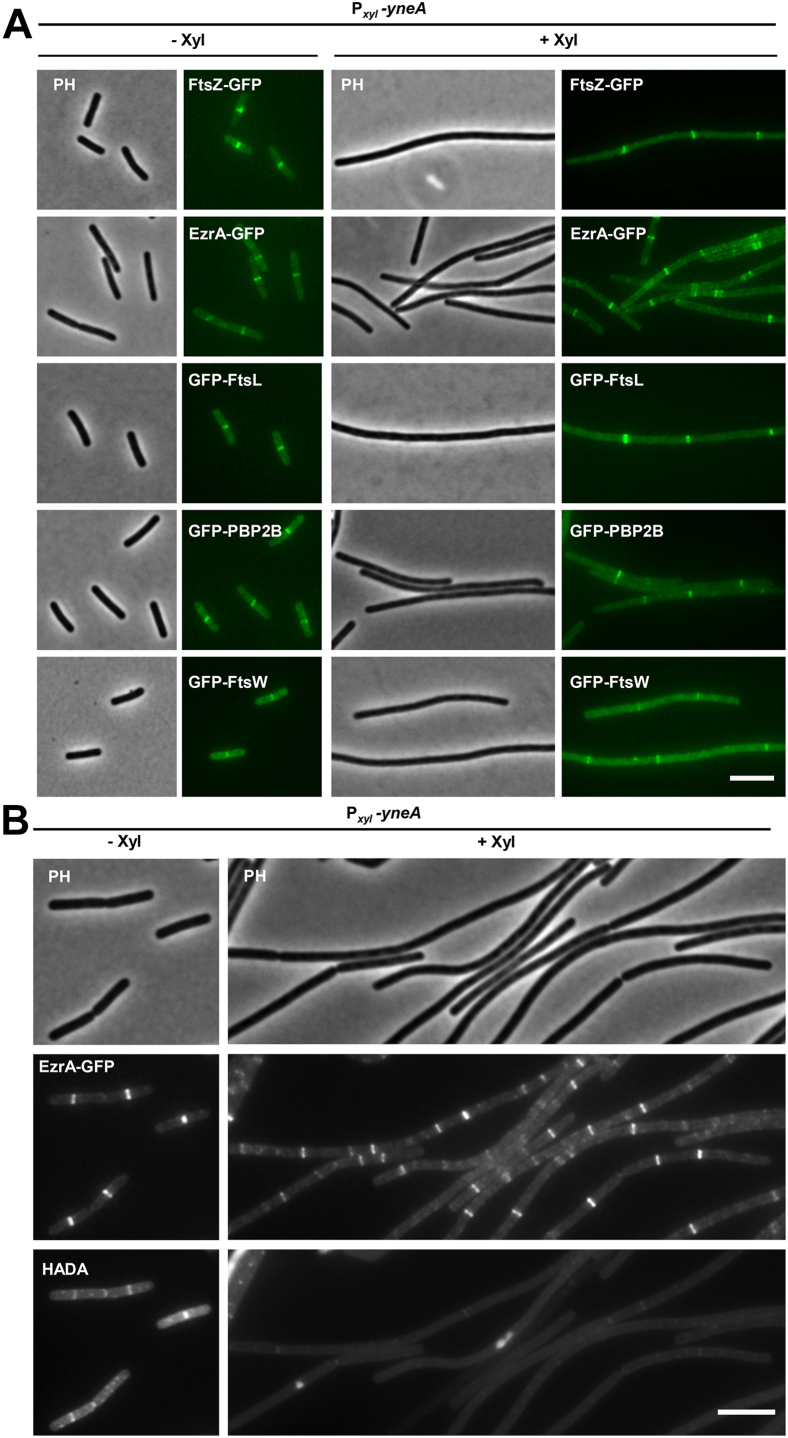
**YneA overexpression blocks septal peptidoglycan synthesis but not divisome assembly**. *A*, representative images of divisome protein localization (FtsZ, EzrA, FtsL, PBP2B and FtsW) in cells with or without YneA overexpression. The localization was assessed in strains 874 (168, *ftsZ::spec ftsZ-gfp*), GYQ28 (168, *ezrA::kan ezrA*-*gfp*), GYQ203 (168, *aprE::erm* P_*spac*_*::gfp-ftsL*), GYQ73 (168, *aprE::erm* P_*spac*_*-gfp-pbp2B*) and GYQ81 (168, *aprE::erm* P_*spac*_*-gfp-ftsW*) carrying plasmid pYW3 (pYW1-*yneA*) (51). These strains were grown at 37 °C in exponential phase. The expression of GFP-FtsL, GFP-PBP2B and GFP-FtsW was induced with 200 μM IPTG, while YneA was induced with 1.5% Xylose for 2 h before imaging. *B*, representative images of EzrA-GFP (marker for Z ring) or HADA (marker for sPG) in cells with or without YneA overexpression. The strain GYQ28 carrying plasmid pYW48 (pHT01-*yneA*) was grown at 37 °C in exponential phase in LB medium without or with 20 μM IPTG to induce YneA overexpression. 2 h post induction, cells were labeled with HADA and then immobilized on 2% agarose pads for photography. The details about HADA labeling are described in Experimental Procedures. Quantification of the co-localization of EzrA-GFP rings and HADA bands in (*B*) is provided in Figure S1*B*. Three biological replicates were carried out for each test. Scale bars, 5 μm.

YneA is a potent cell division inhibitor, but it is rapidly degraded by two membrane-bound proteases, CtpA and DdcP (45). As a result, overexpression of YneA from a xylose-inducible promoter on a plasmid was unable to completely block colony formation under our test conditions (Fig. S1*A*). To circumvent this problem, we investigated the mechanism of YneA by removing one of the proteases (*ctpA* deletion strain) so that its overexpression blocked cell growth and division effectively (Fig. S1*A*). We first checked the localizations of FtsZ and EzrA, which are markers for the Z ring, and FtsL/FtsW/PBP2B, which serve as proxies for the fully assembled divisome, upon YneA overexpression. We expressed YneA in strains carrying fluorescent fusions at the native locus of the respective gene (EzrA-GFP) or under the control of an IPTG-inducible promoter at the *aprE* locus (FtsZ-GFP/GFP-FtsL/GFP-PBP2B/GFP-FtsW) (51). YneA overexpression resulted in a severe division block, but both early (FtsZ, EzrA) and late division proteins (FtsL, FtsW and PBP2B) still localized to the presumptive division sites in cell filaments (Fig. 1*A* and Table S1), suggesting that YneA does not prevent divisome assembly but inhibits its activity.

To determine whether YneA overexpression blocks sPG synthesis, we checked the incorporation of the fluorescent D-amino acid (FDAA) HADA, which has been widely used to label newly synthesized and remodeled PG (52). YneA was expressed under the control of an IPTG-inducible promoter from a plasmid in a strain with EzrA-GFP as the marker for presumptive division sites. As shown in Figure 1*B* and S1*B*, bright HADA bands were observed at approximately 75.14 ± 1.25% of the division sites marked by EzrA-GFP in the absence of YneA overexpression. However, HADA bands were absent at most of the EzrA-GFP bands in cells overexpressing YneA (co-localization rate 11.67 ± 0.58%), indicating that YneA overexpression strongly inhibits sPG synthesis. This is consistent with a recent report that suggests YneA inhibits septal cell wall synthesis (48), however, distinct from the previous report, YneA does not seem to interfere with divisome assembly.

### The transmembrane and LysM domains of YneA are both required for it to inhibit cell division

**Figure 2:**
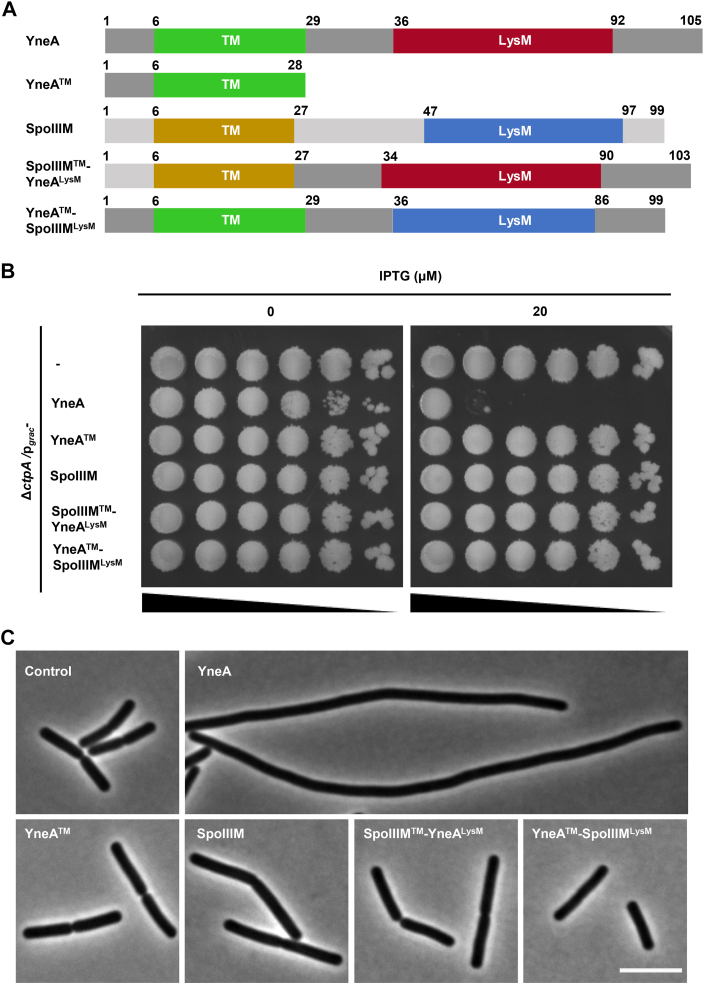
**Both the transmembrane and LysM domains of YneA are critical for its cell division inhibitory activity**. *A*, schematics of the YneA-WT, YneA^TM^ (a truncated form of YneA lacking the LysM domain and C-terminal tail), SpoIIIM-WT, SpoIIIM^TM^-YneA^LysM^ (the TM domain of YneA is replaced with TM domain of SpoIIIM) and YneA^TM^-SpoIIIM^LysM^ (the LysM domain of YneA is replaced with the LysM domain of SpoIIIM). *B*, spot test of the cell division inhibitory activity of proteins indicated in (*A*). Plasmid pHT01K, pYW26 (pHT01K-*spoIIIM*), pYW12 (pHT01K-*yneA*) or its derivatives carrying *yneA* mutations were transformed into strain YW3 (168, *ΔctpA*) on LB plates. Each resulting strain was grown at 37 °C in exponential phase, serially diluted and spotted on LB plates with or without 20 μM IPTG to induce YneA overexpression. Plates were incubated at 37 °C overnight and photographed. *C*, cell morphology of the strains from (*B*). Each strain indicated in (*B*) was grown at 37 °C in exponential phase in LB medium with 20 μM IPTG to induce the expression of YneA or its mutants for 2 h. Cells were immobilized on 2% agarose pads for photography. Three biological replicates were carried out for each test. Scale bar, 5 μm.

Previous studies showed that mutations in the TM domain or LysM domain of YneA reduced or abolished its cell division inhibitory activity (47, 48), suggesting that both domains are necessary for its function. Moreover, replacement of the LysM domain of YneA with the LysM domain from SafA also inactivated YneA (48). However, their roles are not completely understood so far. To confirm the importance of the two domains, we constructed YneA mutants without the LysM domain or chimeras with the TM domain or LysM domain replaced with that of SpoIIIM, a LysM domain-containing protein involved in sporulation of *B. subtilis* with a similar topology to YneA (Fig. 2*A*). Overexpression of YneA^TM^ (a variant lacking the LysM domain) did not block colony growth or cell division, nor did the chimeras harboring the TM or the LysM domain of SpoIIIM (Fig. 2, *B* and *C*, and Table S2). Western blotting of the chimeras fused with GFP revealed that their levels were comparable to that of GFP-YneA (Fig. S2), indicating that the difference in toxicity was not due to variation in protein levels. Thus, both the TM domain and the LysM domain of YneA are critical for its function as previously suggested.

### YneA localizes to the division site *via* its transmembrane domain

**Figure 3:**
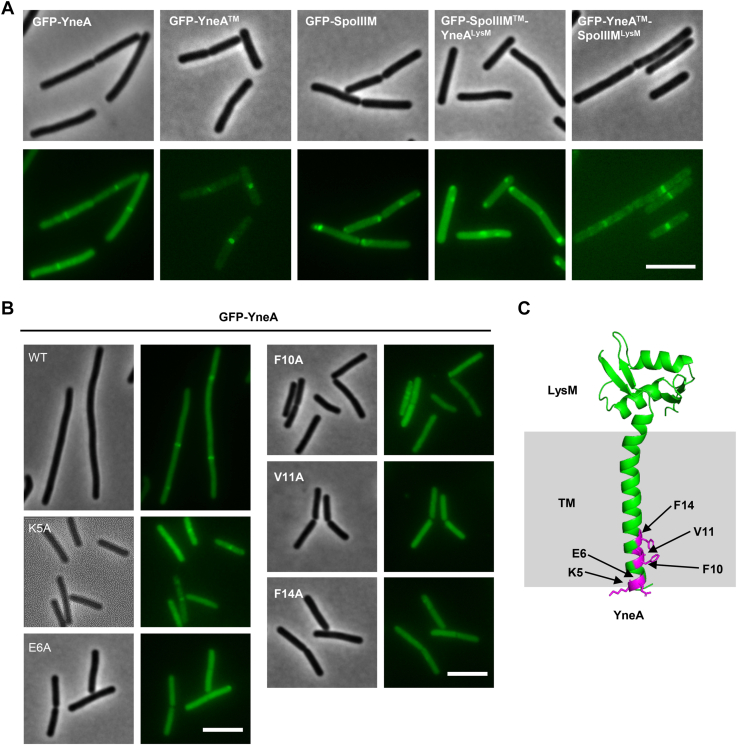
**The transmembrane domain of YneA is critical for its localization and toxicity.***A*, representative images of localization of YneA and its variants. Plasmid pYW29 (pYW1-*gfp*-*spoIIIM*), pYW5 (pYW1-*gfp*-*yneA*) or its derivatives carrying *yneA* mutations were transformed into strain YW3 (168, *ΔctpA*) on LB plates. The resulting strains were grown at 37 °C in exponential phase, GFP fusion proteins were induced with 0.5% Xylose for 2 h before imaging. *B*, representative images of YneA mutant localization. The localization of YneA or its mutants was tested in strain YW3 carrying plasmid pYW5 (pYW1-*gfp-yneA*) or its derivatives containing *yneA* mutations. These strains were grown at 37 °C in exponential phase in LB medium with 0.5% Xylose to induce the expression of GFP fusion proteins for 2 h. Cells were then immobilized on 2% agarose pads for photography. *C*, residues critical for YneA midcell localization and toxicity in a YneA structure (pTM = 0.53) predicted by AlphaFold 3. YneA is colored *green* and critical residues are colored *magenta*. Three biological replicates were carried out for each test. Scale bars in (*A*) and (*B*), 5 μm.

To further test how the TM domain and LysM domain of YneA contribute to its activity, we tested if either was necessary for its midcell localization as a previous study reported that YneA localized to the midcell faintly (48). Consistent with the previous study, we found that GFP-YneA co-localized well with mCherry-ZapA, a Z ring marker (Fig. S3 and Table S3). Interestingly, both GFP-YneA^TM^ and the chimera containing the LysM domain of SpoIIIM (GFP-YneA^TM^-SpoIIIM^LysM^) localized faintly to the midcell, whereas the chimera containing the TM domain of SpoIIIM (GFP-SpoIIIM^TM^-YneA^LysM^) localized to the polar region, behaving similarly to GFP-SpoIIIM (Fig. 3*A* and Table S4). This result indicates that the TM domain of YneA is necessary and sufficient for it to localize to the divisome, while the LysM domain is not.

To further examine the function of the TM domain of YneA, we carried out an alanine-scanning mutagenesis and checked the effect of the mutations on its toxicity. Nine mutations substantially reduced the toxicity of YneA overexpression in the spot test (Fig. S4*A*). Among them, five mutations almost completely eliminated the toxicity of YneA (K5A, E6A, F10A, V11A, and F14A). Western blotting of the mutants fused to GFP showed that most of the mutations did not affect the protein level except for E6A, which markedly increased the level of GFP-YneA for an unknown reason (Fig. S4*B*). Examination of the cell morphology showed that while overexpression of YneA completely blocked cell division, the loss-of-function mutants had little or only a modest impact on cell division (Fig. S4*C*). Most importantly, these YneA mutants (fused to GFP) no longer localized to midcell, but appeared to be evenly distributed in the cytoplasm (Fig. 3*B* and Table S5), suggesting that they disrupt the ability of YneA to target the divisome. Taken together, these analyses demonstrate that the TM domain of YneA is critical for both its localization to midcell and its toxicity.

### Peptidoglycan binding activity of the LysM domain is critical for the cell division inhibitory activity of YneA

**Figure 4:**
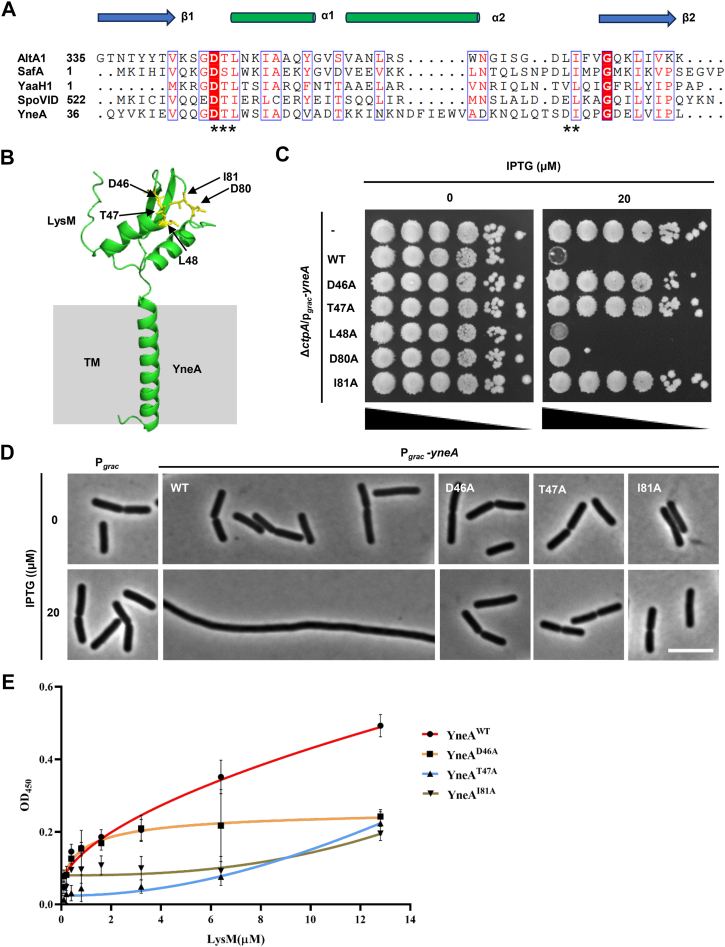
**The peptidoglycan binding ability of the LysM domain of YneA is critical for its activity.***A*, alignment of the LysM domains of YneA and the indicated proteins. Amino acid sequences were obtained from NCBI, aligned with Clustal Omega and displayed with Esprit 3.0. AltA1 is an autolysin from *Enterococcus faecalis*; SafA, YaaH1, SpoVID and YneA are from *B. subtilis*. Residues colored or shaded in *red* represent conserved residues. Five residues marked with ∗ in the LysM domain of YneA were substituted by alanine in this study. *B*, residues substituted by alanine in (*A*) were displayed in a YneA (pTM = 0.53) structural model predicted by AlphaFold 3. YneA is colored *green* and residues substituted are colored *yellow*. *C*, spot test of the cell division inhibitory activity of YneA mutants. Plasmid pHT01 K, pYW12 (pHT01K-*yneA*) or its derivatives carrying *yneA* mutations were transformed into strain YW3 (168, *ΔctpA*) on LB plates. The test was done as in Figure 2*B*. *D*, cell morphology of strains indicated in (*C*). Each strain indicated in (*C*) was grown at 37 °C in exponential phase in LB medium with or without 20 μM IPTG to induce the overexpression of YneA or its mutants for 2 h. Cells were immobilized on 2% agarose pads for photography. *E*, enzyme-linked immunosorbent assay (ELISA) to test the ability of the LysM domain of YneA or mutant forms to bind PG *in vitro*. Details about the ELISA assay were described in Experimental procedures. Three biological replicates were carried out for each test. Scale bar, 5 μm.

The LysM domain is a widely distributed and well-characterized PG binding motif. A previous study identified several conserved residues in the LysM domain of AltA, an autolysin involved in cell division in *Enterococcus faecalis*, which are critical for its affinity for PG (50). To test if the PG binding activity of YneA was critical for its toxicity, we substituted residues in the LysM domain of YneA that were predicted to be important for PG binding based on an alignment of multiple LysM-domain containing proteins (Fig. 4, *A* and *B*), followed by testing their impact on division. While overexpression of YneA blocked colony formation on plates containing 20 μM IPTG, cells overexpressing either one of three mutants (D46A, T47A, or I81A) grew well and divided normally in liquid culture (Fig. 4, *C* and *D* and Table S6). Western blot of GFP fusions to YneA and its mutants showed that their levels were comparable except for YneA-I81A (Fig. S5*A*), suggesting that the lack of toxicity was not due to decreased protein levels but a loss of the ability of the LysM domain to bind PG. In line with this, these mutations reduced the ability of the LysM domain of YneA to bind PG *in vitro* by using an ELISA assay (Fig. 4*E*). However, GFP-YneA mutants harboring any one of these mutations still localized to the division site (Fig. S5*B* and Table S7), confirming that PG binding is not critical for YneA to localize to the divisome. Overall, these results suggest that YneA interacts with the divisome *via* its TM domain so that its LysM domain is positioned at the right place to block sPG synthesis.

### Mutations in FtsW counteract the cell division inhibitory activity of YneA

**Figure 5:**
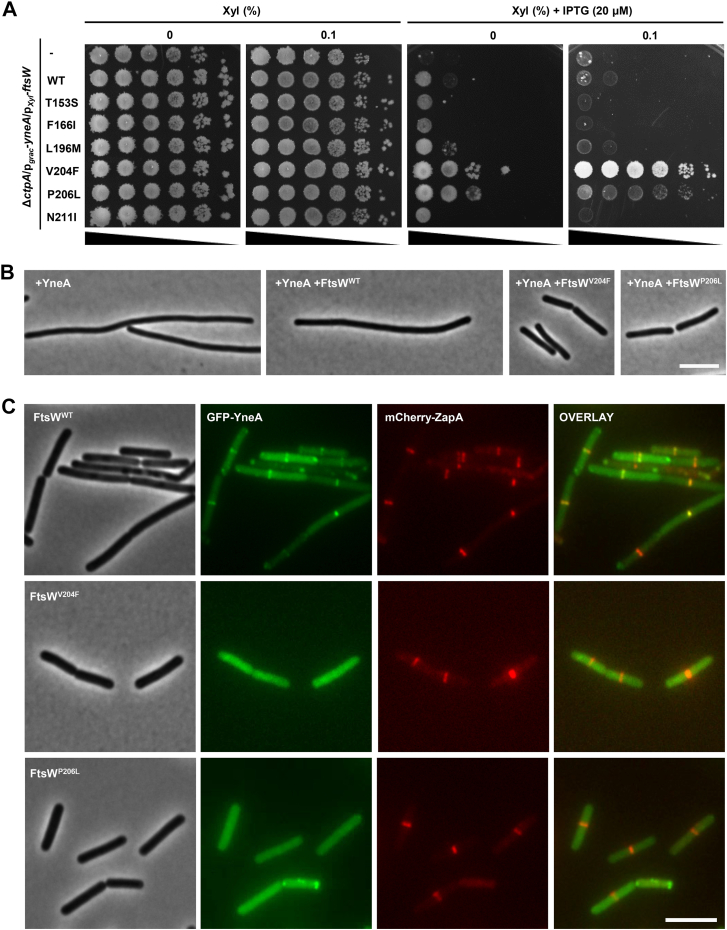
**Mutations in *ftsW* counteract the cell division inhibitory activity of YneA.***A*, spot test of the ability of FtsW mutants to suppress the toxicity of YneA overexpression. Plasmid pYW1, pYW123 (pYW1-*ftsW*) or its derivatives carrying *ftsW* mutations were transformed into strain YW13 [168, *ΔctpA*/pYW12 (pHT01K*-yneA*)] on LB plates. Each resulting strain was grown at 37 °C in exponential phase, serially diluted and spotted on LB plates with or without 20 μM IPTG to induce YneA overexpression, with or without 0.1% Xylose to induce the expression of FtsW or its mutants. Plates were incubated at 37 °C overnight and photographed. *B*, cell morphology of some strains from (*A*). Strains were grown at 37 °C in exponential phase in LB medium with 20 μM IPTG to induce the overexpression of YneA and 0.1% Xylose to induce the expression of FtsW-WT or FtsW mutants for 2 h. Cells were then immobilized on 2% agarose pads for photography. *C*, representative images of GFP-YneA and mCherry-ZapA localization in wild type or FtsW mutant cells. Plasmid pYW5 (pYW1-*gfp*-*yneA*) was transformed into strains YW3 (168, *ΔctpA*), YW149 (168, *ΔctpA ftsW*^*V204F*^) and YW150 (168, *ΔctpA ftsW*^*P206L*^) carrying plasmid pYW10 (pHT01K-*mcherry*-*zapA*). These resulting strains were grown at 37 °C in exponential phase. 0.5% Xylose and 20 μM IPTG were added to induce the expression of GFP-YneA and mCherry-ZapA, respectively, for 2 h before imaging. Three biological replicates were carried out for each test. Scale bars in (*B*) and (*C*), 5 μm.

sPG synthesis in *B. subtilis* is carried out by the synthetic complex consisting of FtsW-PBP2B-DivIB-FtsL-DivIC (equivalent to FtsWIQLB in *E. coli*). Among them, FtsW and PBP2B are a SEDS family PG polymerase and a class B penicillin binding protein (bPBP) with transpeptidase activity, respectively, and are widely conserved among bacteria (53, 54, 55, 56, 57). YneA likely interacts with the sPG synthetic complex to localize to the division site and prevent sPG synthesis. We reasoned that mutations in the primary target of YneA that disrupt their interaction should confer resistance to YneA and detailed characterization of such mutations would provide insights into its working mechanism. YneA has been shown to interact with FtsL and PBP2B, but not FtsW, in a BTH test (48). However, mutations in FtsW, rather than in FtsL or PBP2B, provide resistance to YneA (48). To determine which component of the sPG synthetic complex is the direct target of YneA, we created individual mutant libraries of FtsW/PBP2B/FtsL/DivIC (DivIB is non-essential under a normal culture condition so it was excluded) and screened for mutants suppressing the toxicity of YneA. To do this, the mutant libraries were transformed into a *ΔctpA* strain (chromosomal copy of the gene to be tested was intact) overexpressing YneA under the control of an IPTG-inducible promoter and resistant colonies selected on plate with 20 μM IPTG. We did not obtain any resistant colonies when transformed with the FtsL, DivIC or PBP2B mutant libraries, however, three colonies were obtained after screening >50,000 transformants with the FtsW mutant library. We validated that the suppression of YneA toxicity was plasmid-linked and determined that each of the isolated plasmids harbored two substitutions in *ftsW*: *ftsW* (*T153S* + *P206L*), *ftsW* (*L196M* + *N211I*), and *ftsW* (*F166I* + *V204F*) (Fig. S6, *A* and *B*). To determine which mutation in these double mutants was responsible for the resistance to YneA, each of them was introduced into the parental plasmid and tested for suppression of YneA toxicity in the Δ*ctpA* strain. As shown in Figure 5, *A* and *B* and Table S8, V204F and P206L, both of which are located in TM7 of FtsW, but none of the other single substitutions, provided resistance to YneA overexpression. Although the double mutant *ftsW* (*L196M* + *N211I*) conferred resistance to YneA, neither of the single mutations could. It is noteworthy that P206L and a different substitution at L196 (L196P) had been isolated in a previous study (mis-numbered as P158L and L148P in the study), but only L196P was found to provide resistance to YneA upon DNA damage induced with mitomycin C (48). We focused on the V204F and P206L mutants in the rest of the study because of their strong resistance to YneA.

### FtsW mutations prevent the midcell localization of YneA

In *C. crescentus*, mutations in FtsW resulting in a constitutively active divisome provide resistance to the DNA damage-inducible cell division inhibitors SidA and DidA (40, 41). These activating FtsW mutations cause cells to initiate division early in the cell cycle, resulting in shorter cells. Although the *ftsW*^*L196P*^ mutation did not result in shorter cells, it was assumed to confer resistance to YneA by hyperactivating the divisome in the earlier study (48). To test if the *ftsW*^*V204F*^ or *ftsW*^*P206L*^ mutation we isolated resulted in a constitutively active divisome, we introduced them into the chromosome at the native locus and measured the cell length of the resulting strains. However, the average cell lengths of the FtsW^V204F^ or FtsW^P206L^ mutant cells were comparable to that of wild type cells (Fig. S7, *A* and *B*). Since none of the YneA-resistant FtsW mutations resulted in shorter cells (typical of activation mutations), we suspected that the claim that *ftsW*^*L196P*^ hyperactivates the divisome may be misinterpreted.

To investigate why the FtsW mutations suppressed the toxicity of YneA overexpression, we checked if they prevented the midcell localization of YneA. Strikingly, GFP-YneA was evenly distributed in the cytoplasm in cells expressing FtsW^V204F^ or FtsW^P206L^, whereas it co-localized well with mCherry-ZapA at midcell in cells expressing FtsW (Fig. 5*C* and Table S9). We also found that GFP-YneA failed to localize to midcell in cells expressing FtsW^L196P^ (Fig. S7*C*). These results suggest that YneA depends on FtsW to localize to the division site and the resistant mutations in FtsW prevent YneA recruitment to the divisome, eliminating the toxicity of YneA. In agreement with this, GFP-YneA failed to localize to the presumptive division sites in FtsW-depleted cells but not in FtsL-depleted cells (Fig. S8). Taken together, these results suggest that YneA may interact with FtsW to localize to the divisome to block sPG synthesis.

### YneA interacts directly with FtsW to inhibit cell division

**Figure 6:**
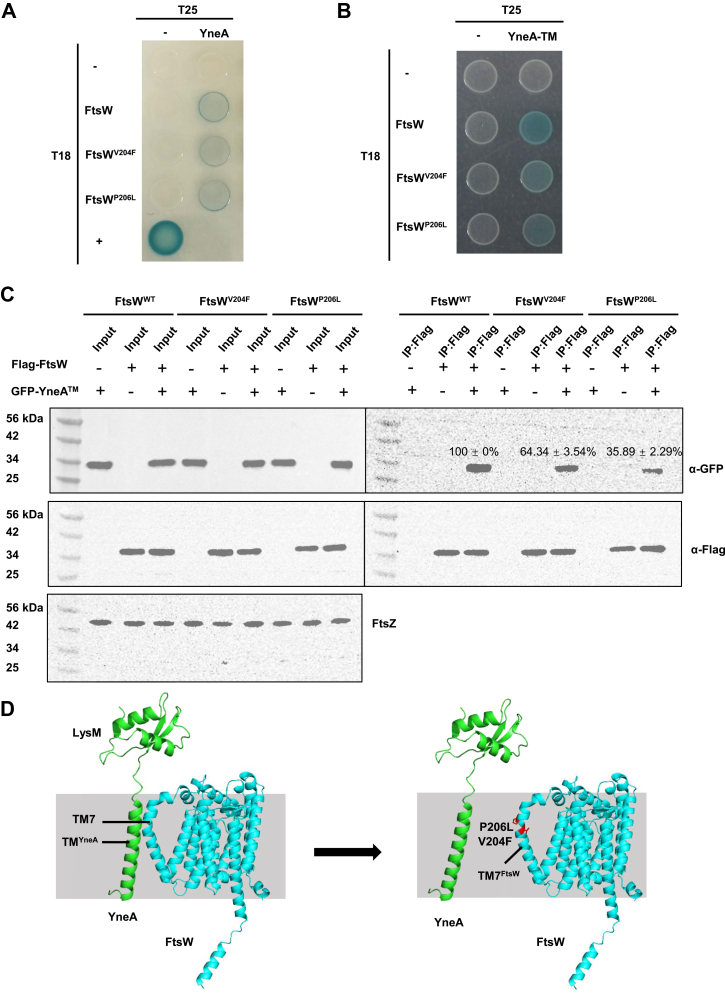
**YneA interacts directly with FtsW.***A*, bacterial two hybrid test of the interaction between YneA and FtsW or its mutants. T18 and T25 fusion plasmids were co-transformed into strain BTH101 on LB plates with antibiotics. Single transformant of each resulting strain was resuspended in 200 μl LB medium and 2 μl of each sample was spotted on LB plates containing antibiotics, 80 μg/mL X-gal and 500 μM IPTG. Plates were incubated at 30 °C for 24 h before analysis. *B*, bacterial two hybrid test of the interaction between YneA^TM^ and FtsW or its mutants. The test was performed as in (*A*). *C*, Co-IP assay to test the interaction between YneA^TM^ and FtsW or its mutants. Plasmid pYW15 (pHT01K-*gfp*-*yneA^TM^*), pYW427 (pYW1-*flag*-*ftsW*) or its derivatives carrying *ftsW* mutations were transformed into strains YW3 (168, *ΔctpA*), YW149 (168, *ΔctpA ftsW*^*V204F*^), and YW150 (168, *ΔctpA ftsW*^*P206L*^). The resulting strains were grown in LB medium at 37 °C, proteins were induced with 0.2% Xylose and 20 μM IPTG. Samples were prepared as described in Experimental procedures and analyzed by Western blot. Input represents the supernatant of cell lysates and IP:Flag indicates the immunocomplexes eluted from the Anti-Flag Nanoab Magnetic Beads after incubation. Three biological replicates were carried out and a representative gel image was shown. The intensity of bands was analyzed in Fiji and normalized to that of wild type FtsW. The average and standard deviation from three repeats were shown. *D*, model for the interference of suppressor mutations *V204F* and *P206L* with the interaction between YneA and FtsW. Structural models of YneA (pTM = 0.53) and FtsW (pTM = 0.93) are predicted by AlphaFold 3, YneA is colored *green*, while FtsW is colored *cyan*. Residues V204F and P206L of FtsW are colored *red*. This model was based on our data, not a structural model of the YneA-FtsW complex.

To test if there is an interaction between YneA and FtsW, we employed the BTH assay (58). We observed a weak interaction signal between YneA and FtsW (Fig. 6*A*). However, the YneA-resistant FtsW mutations did not reduce the interaction signal visually for unknown reasons. Since the TM domain of YneA is necessary and sufficient for its localization to the division site, we tested if YneA interacts with FtsW *via* its TM domain and if the mutations have an observable impact. We detected a modest interaction signal between YneA^TM^ and FtsW and the introduction of either the V204F or P206L mutation into FtsW reduced the interaction signal qualitatively (Fig. 6*B*). In another approach, we used the tripartite Split-FP (fluorescent protein) assay to examine the interaction between YneA^TM^ and FtsW, which is based on the reconstitution of the superfolder GFP (sGFP) when the two proteins of interest interact (Fig. S9, *A* and *B*) (59). We found that when both proteins were fused to the assay tags, sGFP was reconstituted and localized to midcell (Fig. S9*B*). In contrast, no septal localization and only background fluorescence were observed when only one of them was tagged or when FtsW^V204F^ or FtsW^P206L^ was tagged along with YneA (Fig. S9*B*).

To further validate the interaction between YneA^TM^ and FtsW, we conducted Co-immunoprecipitation experiments in cells co-expressing GFP tagged YneA^TM^ (GFP-YneA^TM^) and Flag tagged FtsW (Flag-FtsW). As expected, GFP-YneA co-precipitated with Flag-FtsW in the immunocomplexes with anti-Flag antibodies (Fig. 6*C*). Moreover, the V204F or P206L mutation substantially reduced the amount of GFP-YneA^TM^ in the immunoprecipitates. Taken together, these results indicate that the TM domain of YneA interacts with TM7 of FtsW and the mutations reduce this interaction (Fig. 6*D*).

### Structural model of YneA in complex with the sPG synthetic complex suggests it interacts with FtsW

**Figure 7:**
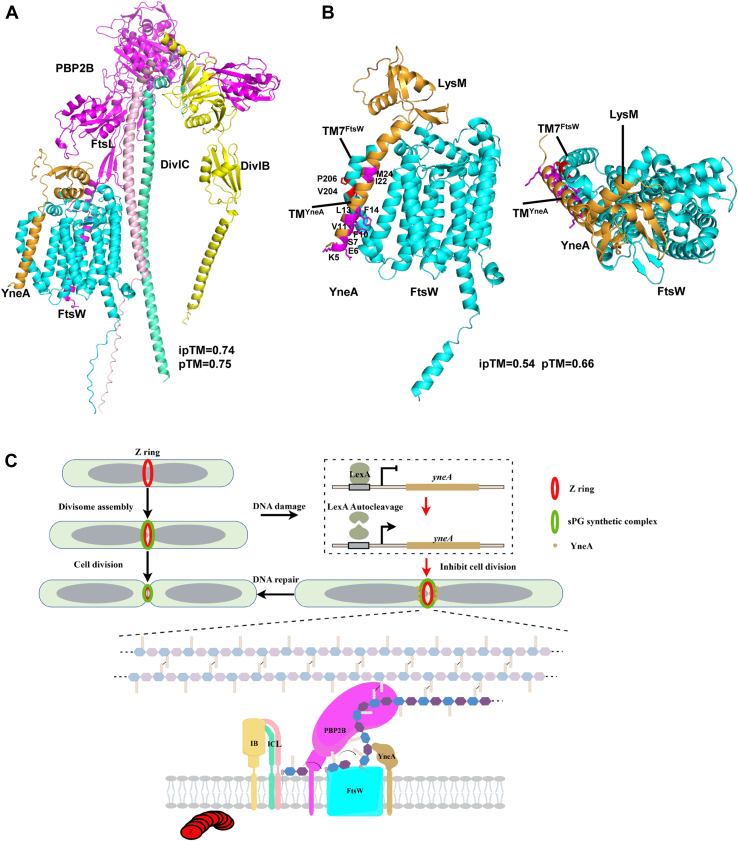
**Structural model of YneA in complex with the sPG synthetic complex and its working model.***A*, a structural model of a multi-protein complex consisting of YneA and the sPG synthetic complex (FtsW-PBP2B-DivIB-FtsL-DivIC; ipTM = 0.74, pTM = 0.75) predicted by AlphaFold 3. YneA, FtsL, DivIC, DivIB, FtsW and PBP2B are colored *bright orange*, *light pink*, *green cyan*, *yellow*, *cyan* and *magenta*, respectively. *B*, structural model of the YneA-FtsW complex (ipTM = 0.54, pTM = 0.66) predicted by AlphaFold 3 and the locations of residues critical for their interaction. YneA and FtsW are colored *bright orange* and *cyan*, V204 and P206 of FtsW and residues important for YneA localization and toxicity are colored *red* and *magenta*, respectively. *C*, a working model for YneA inhibition of cell division. YneA is induced following DNA damage. It localizes to the division site by interacting with FtsW through an interaction between its transmembrane domain (TM) and TM7 of FtsW. The LysM domain of YneA is placed on top of the catalytic cavity of FtsW, binding to the newly synthesized glycan strand generated by FtsW and preventing its elongation or crosslinking into the PG network by PBP2B. The red and green circle stand for the Z ring and sPG synthetic complex, respectively. YneA is depicted as a *bright orange* sphere. The proteins are colored as in (*A*) in the cartoon.

To further investigate the mechanism of YneA, we used AlphaFold 3 to predict the structure of YneA in complex with the sPG synthetic complex FtsW-PBP2B-DivIB-FtsL-DivIC (FtsWIQLB). The predicted structure of the complex (ipTM 0.74; pTM 0.75) indicated that the TM domain of YneA interacts with TM7 of FtsW, but not the other components of the sPG synthetic complex (Fig. 7*A* and Fig. S10*A*). Interestingly, the LysM domain of YneA sits on top of the putative catalytic cavity of FtsW and interacts with the pedestal domain of PBP2B in the structural model of the complex, implying that it may interfere with the catalytic activity of FtsW or the interaction between FtsW and PBP2B. AF3-multimer predictions using just FtsW and YneA also showed that the TM domain of YneA interacts with TM7 of FtsW (ipTM = 0.54, pTM = 0.66) (Fig. S10*B*). Importantly, residues in the TM domain of YneA that are necessary for its localization and toxicity are predicted to interact with FtsW (Fig. 7*B*). When V204F or P206L was introduced into FtsW, the confidence of the structural model of YneA–FtsW complex decreased dramatically and the TM domain of YneA was located away from TM7 of FtsW (Fig. S10, *C* and *D*). This analysis lends additional support to our finding that YneA binds directly to FtsW in the membrane plane to inhibit its activity through the LysM domain.

## Discussion

DNA damage in bacteria induces the SOS response, which commonly upregulates inhibitors of cell division to provide the cells with sufficient time for DNA repair before cell division can proceed (3, 5, 8, 9). SulA-mediated inhibition of Z ring formation in *E. coli* has long been considered as the paradigm for the SOS response-induced cell division block (24). However, accumulating evidence indicates that small integral membrane proteins targeting late steps of cell division are more widespread (40, 41, 42, 44, 47, 48), but how these division inhibitors work is not completely understood. In this study, we discover that YneA in *B. subtili*s, a prototype of these small membrane-bound division inhibitors, interacts with the essential SEDS family PG polymerase FtsW through its TM domain and uses its LysM domain to block sPG synthesis (Fig. 7*C*). Structural modeling further supports our working model of YneA. These findings not only reveal the working mechanisms of YneA, but also provide important clues for designing inhibitors of the SEDS family PG polymerases represented by FtsW.

How YneA is upregulated upon DNA damage and how it is removed after DNA damage repair have been well characterized (3). However, the mechanism by which it blocks cell division remains incompletely understood. Previous studies suggested that YneA overexpression likely inhibits cell division by interfering with divisome assembly and preventing septal cell wall synthesis (48). By systemic examination of the localization of early divisome proteins (FtsZ and EzrA) and late ones (FtsL, FtsW and PBP2B), as well as checking sPG synthesis (HADA incorporation), we confirmed that YneA blocks sPG synthesis but not Z ring formation or divisome assembly (Fig. 1). Previous studies have also found that both the TM and LysM domains of YneA are necessary for its division inhibitory activity, but their roles have not been clearly defined (47, 48). We found that the TM domain is necessary and sufficient for YneA to localize to the division site, whereas the LysM domain is not (Fig. 3*A*). As a result, many mutations in the TM domain abolish the midcell localization of YneA and its division inhibitory activity (Fig. 3*B* and Fig. S4), while mutations in the LysM domain or its truncation eliminate YneA toxicity but not its localization to the midcell (Fig. 4 and Fig. S5). Importantly, mutations reducing the LysM domain’s affinity for PG inactivate YneA (Fig. 4), indicating that the cell division inhibitory activity of YneA relies on its PG binding activity. Taken together, these results indicate that YneA uses its TM domain to target the divisome and then inhibits sPG synthesis *via* its LysM domain.

The observations that YneA can localize to the division site and inhibits sPG synthesis indicate that it interacts with the sPG synthetic complex FtsW-PBP2B-DivIB-FtsL-DivC in *B. subtilis*. A previous study showed that YneA interacts with FtsL and PBP2B, but not FtsW, in a BTH test (48). Despite that they isolated mutations in FtsW that are refractory to the cell division inhibitory activity of YneA, they proposed that YneA interacts with FtsL and PBP2B (but not FtsW) to interfere with the assembly of the sPG synthetic complex (48). However, through characterization of YneA-resistant FtsW mutations, we provide compelling evidence that YneA directly interacts with FtsW to inhibit sPG synthesis. Firstly, we detected an interaction between YneA and FtsW by BTH, Split-FP and Co-IP assays, mediated by its TM domain (Figs. 6 and S9). Introduction of either one of the YneA-resistant mutations (*ftsW*^*V204F*^ and *ftsW*^*P206L*^) in FtsW reduced the interaction signal. We do not understand why the previous study failed to detect an interaction between FtsW and YneA, but it could be due to difference in the constructs, strain background, growth conditions, expression levels, or other technical aspects. Secondly, we found that the midcell localization of YneA depends on the presence of FtsW (Fig. S8), and YneA failed to localize to the division site in FtsW^V204F^ or FtsW^P206L^ mutant cells (Fig. 5*C*). We also found that the YneA-resistant mutation FtsW^L196P^ studied in detail in the earlier study prevents YneA from localizing to the divisome (Fig. S7*C*). Finally, an AlphaFold 3 structural model of YneA in complex with the sPG synthetic complex indicates that it interacts with TM7 of FtsW (Fig. S7, *A* and *B*). The predicted interaction interface matches nicely with our genetic, biochemical and imaging results. Thus, we conclude that YneA targets the divisome by binding to FtsW in the membrane plane and then inhibits sPG synthesis through its LysM domain (Fig. 7*C*). It is noteworthy that we could not exclude the possibility that YneA may also interact with PBP2B and FtsL as reported previously (48), but these interactions, if they exist, are likely not that critical for it to inhibit cell division.

An unresolved question in this study is how YneA inhibits the function of FtsW. A recent structural study of the lateral PG synthase RodA-PBP2 complex in *E. coli,* another SEDS-bPBP complex equivalent to FtsW-PBP2B, revealed the mechanism of Lipid II polymerization (60). RodA contains two putative substrate binding cavities, one is the acceptor site and the other is the donor site, both of which recognize Lipid II. RodA catalyzes the polymerization of these two Lipid II molecules into Lipid IV, which is then elongated by addition of more Lipid II molecules from the acceptor site. The emerging glycan strand is then crosslinked into the existing cell wall by the transpeptidase PBP2 (60). FtsW and PBP2B form a highly similar structure and is believed to function similarly. YneA binds to TM7 of FtsW, which is one of the TMs constituting the donor site (TM6, TM7, and TM9). One would expect that this binding might interfere with the ability of the donor site to recognize Lipid II, thus preventing PG polymerization by FtsW. However, although the TM domain of YneA alone is sufficient to localize to the division site and to interact with FtsW (Figs. 3*A* and 6), it is unable to block cell division (Fig. 2*B*), suggesting that the interaction between the TM domain of YneA and TM7 of FtsW does not affect its ability to bind to Lipid II. The AlphaFold 3 model of YneA in complex with the sPG synthetic complex suggests that the binding between YneA and FtsW would place its LysM domain on top of the catalytic cavity of FtsW in the periplasm (Fig. 7*A*). Thus, one possibility is that the LysM domain of YneA inhibits activation of the glycosyltransferase activity of FtsW by directly affecting the catalytic site or interfering with FtsW’s interaction with PBP2B. However, several lines of evidence suggest that this is not the case. First, we did not isolate mutations in FtsW that result in a constitutively active divisome. Instead, only mutations in TM7 of FtsW that disrupt its interaction with YneA were obtained. Second, LysM domain’s ability to bind PG is critical for the cell division inhibitory activity of YneA (Fig. 4). Although the LysM domain also appears to interact with PBP2B in the AF 3 model, the residues important for PG binding and cellular toxicity are not in the putative interface (Fig. S11). Also, we did not isolate PBP2B mutants resistant to YneA overexpression. Thus, we prefer that the LysM domain binds to PG, leading to inhibition of cell division. We speculate that once YneA binds to FtsW, its LysM domain binds to the emerging glycan strand synthesized by FtsW, preventing the extension of the glycan strand (Fig. 7*C*).

We tried to verify the above model by *in vitro* PG polymerization assay using purified FtsW-PBP2B and YneA. However, purified FtsW-PBP2B did not exhibit polymerase activity (Fig. S12), and attempts to purify the FtsW-PBP2B-FtsL-DivIC-DivIB complex were not successful so far. Thus, future efforts to establish an *in vitro* PG polymerization assay of the FtsW-PBP2B complex and characterization of the effect of YneA on the activity of the complex will be key to elucidate the mechanism of action of YneA.

In addition to YneA of *B. subtilis*, many bacteria also employ small integral membrane proteins to inhibit late steps of cell division upon DNA damage. We wondered if these cell division inhibitors also interact with FtsW to inhibit sPG synthesis. Therefore, we generated AlphaFold 3 models for these cell division inhibitors in complex with the sPG synthetic complex FtsWIQLB of their respective organisms, including ChiZ from *M. tuberculosis,* SosA from *S. aureus* and SidA from *C. crescentus*. Interestingly, all these cell division inhibitors were predicted to bind to FtsW rather than the other components of the sPG synthetic complex, albeit the models were not of high confidence (Figs. S13 and 14). AF3-multimer predictions of these division inhibitors with FtsW further suggested that their TM domain interacts with TM7 of FtsW (Fig. S15). ChiZ, a LysM-domain containing protein with a similar topology of YneA, likely inhibits cell division *via* a similar mechanism as YneA since it is predicted to bind to TM7 of *Mt*FtsW with its LysM domain sitting on top of the catalytic cavity of *Mt*FtsW (Figs. S13*A* and S15*A*). SosA was also predicted to bind TM7 of *Sa*FtsW, but it does not contain a LysM domain (Figs. S13*B* and S15*B*). However, its extended helix may interfere with the catalytic cavity of *Sa*FtsW since a previous study found that the extended region is critical for its toxicity. SidA was also predicted to bind to the TM7 of *Cc*FtsW, however, it seems to bind to the opposite side of TM7 compared to YneA/ChiZ/SosA (Figs. S13*C* and S15*C*). Thus, its mechanism of action may be different from those of YneA/ChiZ and SosA. These analyses imply that inhibition of FtsW function may be a conserved and effective mechanism for DNA damage inducible membrane-bound division inhibitors to coordinate DNA damage repair and cell division. Uncovering the underlying mechanisms of these small division inhibitors may shed lights on the enzymatic mechanism of FtsW and designing its inhibitors.

## Experimental procedures

### Bacterial strains, plasmids, and growth conditions

Strains, plasmids and primers used in this study are listed in Supplementary Materials 2–4 in Supporting Information, respectively. Construction of strains and plasmids is described in detail in Supporting Information with the primers listed in Supplementary Material 4. Cells were grown in Luria-Bertani (LB) medium (1% tryptone, 0.5% yeast extract and 0.5% NaCl) at indicated temperatures. Antibiotics were used at the following concentrations: ampicillin = 100 μg/ml; kanamycin = 25 μg/ml; tetracycline = 25 μg/ml; erythromycin = 5 μg/ml and chloramphenicol = 7.5 μg/ml. Xylose and IPTG were used at appropriate concentrations to induce the expression of proteins.

### Bacterial two hybrid assay

To detect the interaction between proteins, plasmids expressing the T18 and T25 fusions of indicated proteins were co-transformed into strain BTH101(58). The co-transformants were selected on LB plates containing 100 μg/ml ampicillin and 25 μg/ml kanamycin at 30 °C for 36 h. Single transformants were resuspended in 200 μl LB medium and 2 μl of each sample was spotted on LB plates containing 100 μg/ml ampicillin, 25 μg/ml kanamycin, 80 μg/ml X-gal and 500 μM IPTG. Plates were incubated at 30 °C for 24 h before analysis.

### Spot titer assays

Overnight cultures of *B. subtilis* strains were diluted 1:100 in fresh LB medium with antibiotics, and grown at 37 °C in exponential phase. The cultures were serially diluted and 2 μl of each dilution was spotted on LB plates with antibiotics, with or without IPTG or Xylose at the indicated concentration. Plates were incubated at 37 °C overnight and photographed.

### Fluorescence microscopy

All phase contrast and fluorescence images were acquired using an Olympus BX53 upright microscope with a Retiga R1 camera from QImaging, a CoolLED pE-4000 light source and a U Plan X Apochromat phase contrast objective lens (100 × , 1.45 numerical aperture [NA], oil immersion). The fluorescence images of Green, mCherry and HADA were obtained using the Chroma EGFP filter set EGFP/49002, mCherry/Texas Red filter set mCherry/49008 and the DAPI filter set DAPI/49000, respectively.

#### Localization of GFP-YneA and its mutants

Overnight cultures of strain YW3 (168, *ΔctpA*) carrying plasmid pYW5 (P_*xyl*_::*gfp*-*yneA*) or pYW13 (P_*grac*_::*gfp*-*yneA*) or its derivatives were diluted 1:100 in fresh LB medium with antibiotics, and grown at 37 °C to OD_600_ ∼ 0.5. The cultures were then diluted 1:10 in fresh LB medium with antibiotics and 0.5% Xylose or 10 μM IPTG, and grown at 37 °C for 2 h. Cells were immobilized on 2% agarose pads for photography.

#### Co-localization of GFP-YneA and mCherry-ZapA

Overnight cultures of strains YW3 (168, *ΔctpA*), YW149 (168, *ΔctpA ftsW*^*V204F*^), or YW150 (168, *ΔctpA ftsW*^*P206L*^) carrying plasmid pYW5 (P_*xyl*_-*gfp*-*yneA*) and pYW10 (P_*grac*_::*mCherry*-*zapA*) were diluted 1:100 in fresh LB medium with antibiotics, and grown at 37 °C to OD_600_ ∼ 0.5. The cultures were then diluted 1:10 in fresh LB medium with antibiotics, 0.5% Xylose and 20 μM IPTG, and grown at 37 °C for 2 h. Cells were immobilized on 2% agarose pads for photography.

#### Localization of FtsZ, EzrA, FtsL, PBP2B, and FtsW upon YneA overexpression

To detect whether YneA overexpression disrupt divisome assembly, overnight cultures of strains 874 (168, *ftsZ::spec ftsZ*-*gfp*), GYQ28 (168, *ezrA::kan ezrA*-*gfp*), GYQ203 (168, *aprE::erm* P_*spac*_::*gfp-ftsL*), GYQ73 (168, *aprE::erm* P_*spac*_::*gfp-pbp2B*) and GYQ81(168, *aprE::erm* P_*spac*_::*gfp-ftsW*) carrying plasmid pYW3 (P_*xyl*_::*yneA*) were diluted 1:100 in fresh LB medium with antibiotics, and grown at 37 °C to OD_600_ ∼ 0.5. The cultures were then diluted 1:10 in fresh LB medium with antibiotics, 200 μM IPTG (without IPTG for strain 874 and GYQ28) and with or without 1.5% Xylose, grown at 37 °C for 2 h. Cells were immobilized on 2% agarose pads for photography.

#### Localization dependency of YneA

To test if the localization of YneA at the division site depends on FtsL or FtsW, overnight cultures of strains YW94 (168, *aprE::erm* P_*spac*_*::gfp*-*ftsL ftsL*_*native*_*::*P_43_ *ΔctpA*) and YW109 (168, *aprE::erm* P_*spac*_*::gfp-ftsW ΔctpA ΔftsW*_*native*_) carrying plasmid pYW5 (P_*xyl*_*::gfp*-*yneA*) were diluted 1:100 in fresh LB medium with antibiotics and 50 μM IPTG, grown at 37 °C to OD_600_ ∼ 0.5. Cells were then collected by centrifugation and washed twice with fresh LB medium to remove the IPTG, followed by resuspension in the same volume of LB medium. These cultures were then diluted 1:25 in fresh LB medium with antibiotics, grown at 37 °C. After the removal of IPTG for 2 h, 0.5% Xylose was added to cultures to induce the expression of GFP-YneA for another 2 h before imaging.

#### HADA labeling to detect sPG synthesis

To test if YneA overexpression blocked sPG synthesis which can be labeled by HADA, overnight cultures of strain GYQ28 (168, *ezrA::kan ezrA*-*gfp*) carrying plasmid pHT01 (P_*grac*_) or pYW48 (P_*grac*_::*yneA*) were diluted 1:100 in fresh LB medium with antibiotics, and grown at 37 °C for 2 h. The cultures were then diluted 1:10 in fresh LB medium with antibiotics and 20 μM IPTG. A 200 μl sample was then taken from each culture and incubated with 4 μl of HADA (final concentration, 0.5 mM) in dimethyl sulfoxide (DMSO) at 37 °C for 3 min. After incubation, the cells were immediately fixed with paraformaldehyde and glutaraldehyde for 15 minutes on ice and then washed five times with phosphate-buffered saline (PBS). The cells were resuspended in 50 μl PBS and immobilized on 2% agarose pads for photography. The fluorescent D-amino acid HADA was purchased from the company Scilight-Peptide (http://www.scilight-peptide.com/).

### Purification of His-tagged LysM domain or its mutants

Overnight cultures of *E. coli* strain BL21 (DE3) carrying a plasmid expressing a His-tagged protein were diluted 1:100 in 200 ml LB medium with antibiotics and grown at 37 °C until OD_600_ reached about 0.4. Then 500 μM IPTG was added to cultures to induce protein expression at 16 °C for 12 h. Cells were collected by centrifugation at 4 °C, resuspended in 20 ml pre-cold lysis buffer (50 mM Tris-HCl (pH 8.0), 500 mM NaCl, 5% glycerol, 0.1 mM dithiothreitol and 20 mM imidazole), and then lysed by sonication. The lysates were centrifuged for 10 minutes at 4 °C to remove cell debris. The supernatants were then loaded onto Ni-NTA agarose. The column was washed twice using 20 ml pre-cold washing buffer (50 mM Tris-HCl (pH 8.0), 1 M NaCl, 5% glycerol, 0.1 mM dithiothreitol and 50 mM imidazole) to remove miscellaneous proteins. The bound protein was eluted by elution buffer (50 mM Tris-HCl (pH 8.0), 500 mM NaCl, 5% glycerol, 0.1 mM dithiothreitol and 500 mM imidazole) and analyzed by SDS-PAGE gel. The elution samples with high protein concentration were collected, dialyzed against storage buffer (50 mM Tris-HCl (pH 8.0), 500 mM NaCl, 5% glycerol and 0.1 mM dithiothreitol), concentrated and stored at −80 °C. The protein concentration was measured using the Bradford assay.

### Enzyme-linked immunosorbent assay

To test the binding of the LysM domain to peptidoglycan fragments, the assay was performed as previously described (50). 96-well polystyrene plates were coated with 100 μl peptidoglycan fragments (peptidoglycan was purchased from Sigma-Aldrich: Cat #SMB00288, diluted in PBS to a final concentration of 2 mg/ml and lysed by sonication for 3 min) and incubated at 22 °C for 1 h. The plates were washed three times with PBST (0.25% Tween-20), and blocked with 2% skimmed milk at 22 °C for 2 h. After washed with PBST three times, 100 μl of serially diluted His-LysM^WT^ or its mutants was added to the peptidoglycan-coated plates. After 1 h incubation at 22 °C, the plates were washed with PBST for five times and then incubated with Mouse anti-His antibodies (1/3000) at 22 °C for 1 h. After washed with PBST for five times, the plates were incubated with HRP-conjugated Goat anti-Mouse antibodies (1/3000) at 22 °C for 1 h. Following washed for five times, 100 μl TMB substrate were added to the plates and all reactions were stopped by the addition of stop solution after the same induction time. Finally, the absorbance at 450 nm was measured.

### Western blot

To test the stability of YneA mutants, overnight cultures of strain YW3 (168, *ΔctpA*) carrying plasmid pYW5 (P_*xyl*_::*gfp*-*yneA*) or its derivatives were diluted 1:100 in LB medium with antibiotics and grown at 37 °C until OD_600_ reached about 0.4. Xylose was then added to cultures to a final concentration of 0.5% and incubated at 37 °C for another 2 h. The OD_600_ of cultures was adjusted to 1.0 to ensure the same amounts of cells were taken for analysis. A 1 ml sample was taken from each culture, washed three times with PBS, and then resuspended in 100 μl 1 × SMM buffer (0.5 M sucrose, 0.02 M maleic acid, 0.02 M MgCl_2_, adjusted to pH 6.5) with 1 mg/ml lysozyme and incubated at 37 °C for 1 h. Subsequently, samples were mixed with 6 × SDS-PAGE sample buffer to 1 × and boiled at 95 °C for 10 min. Samples were separated *via* 12% SDS-PAGE gel and transferred onto a nitrocellulose membrane. The membranes were incubated in 5% skimmed milk blocking agent for 1 h at room temperature and then incubated with primary antibodies (anti-GFP, 1:10,000 dilution) at room temperature for 1 h or 4 °C overnight. Primary antibodies were removed, and membranes were washed with fresh TBST three times (10 min/time). Then membranes were incubated with secondary antibodies (1:10,000 dilution) at room temperature for 1 h. After washing, membranes were incubated with Smart-ECL Enhanced for 5 min and imaged.

### Creation of FtsL, DivIC, FtsW, and PBP2B mutant libraries and screen for suppressors of YneA overexpression

#### Creation of FtsL, DivIC, FtsW, and PBP2B mutant libraries

The FtsW mutant library was obtained by random PCR mutagenesis using the primers PHY-*ftsW*-F and PHY-*ftsW*-R and plasmid pYW123 (pYW1-*ftsW*) as template. Mutagenized-PCR product digested with BamHI and EcoRI was ligated into plasmid pYW1 (PHY300-*p*_*xyl*_) digested with the same enzymes. Ligation product was transformed into DH5α competent cells and plated on LB plates with ampicillin. These plates were washed with LB to collect about 30,000 transformants together, and plasmids were isolated and stocked. DivIC, FtsL, and PBP2B mutant libraries were constructed similarly.

#### Screen for suppressors of YneA overexpression

The mutagenized plasmid pYW123 (P_*xyl*_*-ftsW*) was transformed into YW13 (168, *ΔctpA*/P_*grac*_-*yneA*) and plated on LB plates with antibiotics, 20 μM IPTG and 0.1% Xylose at 37 °C for 24 h. YneA overexpression was toxic to YW13 and the expression of FtsW from the plasmid could not rescue the growth defect. Thereby, transformants that could grow on selective plates likely contained plasmids expressing FtsW mutants suppressing the overexpression of YneA. In the screen with the FtsW mutant library, three transformants were picked and plasmids were then isolated and transformed back into strain YW13 (168, *ΔctpA*/P_*grac*_::*yneA*) which had a clean background to validate their ability to suppress YneA overexpression. The *ftsW* gene in suppressor plasmids was then sequenced to identify the mutations. The sequencing result showed that each plasmid contained two mutations and individual mutation was introduced into plasmid pYW123 (P_*xyl*_*::ftsW*) by site-directed mutagenesis and tested for determination of the causative mutation. Two (V204F and P206L) of the six mutations could suppress YneA overexpression. The screens for FtsL, DivIC and PBP2B mutant libraries were performed as that of FtsW, but no resistant transformants were obtained.

### Split-FP assay

To detect the interaction between YneA^TM^ and FtsW or its mutants, we employed the tripartite Split-FP (Fluorescent Protein) assay (59). In this assay, overnight cultures of strains YW3 (168, *ΔctpA*) carrying plasmid pYW424 (P_*grac*_*::sfGFP1-9+sfGFP10-yneA^TM^+ sfGFP11-ftsW*^*WT*^), YW149 (168, *ΔctpA ftsW*^*V204F*^) carrying plasmid pYW425 (P_*grac*_*::sfGFP1-9+sfGFP10-yneA^TM^+ sfGFP11-ftsW*^*V204F*^), and YW150 (168, *ΔctpA ftsW*^*P206L*^) carrying plasmid pYW426 (P_*grac*_*::sfGFP1-9+sfGFP10-yneA^TM^+ sfGFP11-ftsW*^*P206L*^) were diluted 1:100 in LB medium with antibiotics and grown at 37 °C for 2 h until OD_600_ was reached about 0.2. IPTG was then added to cultures to a final concentration of 1 mM, and cultures were incubated at 30 °C for another 3 h. Cells were immobilized on 2% agarose pads for photography.

### Purification of a *Bs*FtsW–PBP2B complex

Overnight cultures of *E. coli* strain C43 (pBAD33-ULPI) carrying a plasmid (pCOLA Duet-*6his*-*sumo*-*flag*-*ftsW*-*pbp2B*) co-expressing a Flag-tagged *Bs*FtsW and untagged *Bs*PBP2B were diluted 1:100 in 2 L TB medium with antibiotics and grown at 37 °C until OD_600_ reached about 0.8. Then 100 μM IPTG and 0.1% L-Arabinose were added to cultures to induce the expression of *Bs*FtsW-PBP2B and ULP1 at 18 °C for 18 h. Cells were collected by centrifugation at 4 °C, resuspended in 250 ml pre-cold lysis buffer (20 mM HEPES (pH 7.5), 300 mM NaCl, 10% glycerol, 20 mM MgCl_2_, 5 mM dithiothreitol and 3 μl Benz-Neburase Benzonase Nuclease), and then lysed by high-pressure homogenization at 800 bar. The lysates were centrifuged at 12,000 rpm for 20 minutes at 4 °C to remove cell debris. The membrane fraction containing the target protein was isolated by centrifugation at 140,000×*g* for 60 minutes at 4 °C, and solubilized in Flag balance buffer (20 mM HEPES (pH 7.0), 500 mM NaCl and 10% glycerol) containing 1% LMNG for 1 h at 4 °C. The mixture was centrifuged at 16,000 rpm for 30 minutes at 4 °C and the supernatant was incubated with Anti-Flag affinity resin for 2 h at 4 °C with shaking. The resulting mixture was then loaded onto a gravity column. The column was washed using 100 ml pre-cold washing buffer (20 mM HEPES (pH 7.0), 500 mM NaCl and 10% glycerol) containing 0.01% LMNG to remove miscellaneous proteins. The bound protein was eluted by elution buffer (20 mM HEPES (pH 7.0), 500 mM NaCl, 10% glycerol, and 0.3 *g* 3×Flag peptide) containing 0.005% LMNG. The elution sample was concentrated, purified by size-exclusion chromatography (SEC) using a Superose 6 Increase 10/300 Gl column, and analyzed by SDS-PAGE gel. The protein concentration was measured using Nanodrop One. The purification of the *Pa*QLBWI complex (*Pseudomonas aeruginosa*) was performed as the *Bs*FtsW-PBP2B complex, except that plasmids pCOLA Duet-*flag*-*PaWI* and pET Duet-*PaftsQBL* were used.

### Examination of the PG polymerase activity of the *Bs*FtsW-PBP2B complex

As previously described (61), the Lipid II substrate was purified from strain PY79 (*B*. *subtilis*). The purified *Bs*FtsW-PBP2B complex was incubated with Lipid II, *Sa*PBP4 and BDL in reaction buffer (200 mM HEPES, pH 7.5 and 20 mM MnCl_2_) at 25 °C for 30 min. Additionally, the *Pa*FtsQBLWI complex was incubated with Lipid II as a positive control. After the reactions, samples were mixed with 6 × SDS-PAGE sample buffer to 1 × and the mixtures were centrifuged at 16,000 rpm for 30 minutes at room temperature to remove insoluble precipitates. Subsequently, samples were separated *via* 4–20% SDS-PAGE gel and transferred onto a nitrocellulose membrane. The membranes were incubated in 5% skimmed milk blocking agent for 1 h at room temperature and then incubated with HRP-conjugated anti-Strep antibodies (1/5000) at 37 °C for 1 h. After six times washes, membranes were incubated with Smart-ECL Enhanced for 5 min and imaged.

### Co-immunoprecipitation (Co-IP)

To detect the interaction between YneA^TM^ and FtsW or its mutants, overnight cultures of strains were diluted 1:100 in 50 mL LB medium with antibiotics and grown at 37 °C for 2 h until OD_600_ reached about 0.2 to 0.4. 0.2% Xylose and 20 μM IPTG were then added to the cultures and incubated at 37 °C for another 2 h. Cells were collected by centrifugation, washed twice with 10 ml pre-cold PBS buffer (0.36 M NaCl, 2.6 mM KCl, 8 mM Na_2_HPO_4_, 2 mM KH_2_PO_4_, pH 7.3) and resuspended in 1 ml pre-cold NP40 lysis buffer (150 mM NaCl, 1.0% Triton X-100, 50 mM Tris, pH 8.0) containing protease inhibitor, and then lysed by sonication. The lysates were centrifuged for 10 minutes at 4 °C to remove cell debris. A 400 μl sample taken from each supernatant was mixed with pre-washed Anti-Flag Nanoab Magnetic Beads (Lablead, https://www.lablead.cn/) and incubated at 4 °C for 12 h. Anti-Flag Nanoab Magnetic Beads-protein complexes were washed four times with PBST buffer (0.36 M NaCl, 2.6 mM KCl, 8 mM Na_2_HPO_4_, 2 mM KH_2_PO_4_, 0.5% Tween-20, pH 7.3), resuspended in 40 μl 1x SDS-PAGE sample buffer and then vortexed vigorously for 2 min to elute immunocomplexes. Samples were separated *via* 10% SDS-PAGE gel and transferred onto a nitrocellulose membrane. The membranes were incubated in 5% skimmed milk blocking agent for 1 h at room temperature, and then incubated with primary antibodies (anti-Flag or anti-GFP, 1:10,000 dilution) at room temperature for 1 h or 4 °C overnight. Primary antibodies were removed, and membranes were washed with fresh TBST three times (10 min/time). Then membranes were incubated with secondary antibodies (1:10,000 dilution) at room temperature for 1 h. After washing, membranes were incubated with Smart-ECL Enhanced for 5 min and imaged. FtsZ serves as a loading control, with sera raised against *E. coli* FtsZ (1/10,000 dilution) which were shown to be able to detect FtsZ from *B. subtilis* previously (62).

## Data availability

All strains and plasmids used in this study are listed in Tables S1 and Supplementary Materials 2 and 3, respectively. All data presented in this study will be publicly available as of the data of publication.

## Supporting information

This article contains supporting information.

## Conflict of interest

The authors declare that they have no conflicts of interest with the contents of this article.
