# Supplementary material for: The SOS response-induced cell division inhibitor YneA interacts with FtsW to block septal peptidoglycan synthesis in Bacillus subtilis
Source: J Biol Chem. 2026 Jun 23;302(8):113276. doi: 10.1016/j.jbc.2026.113276 (PMC13396833; doi:10.1016/j.jbc.2026.113276)
Supplement: Supplementary Material 1 [file mmc1.docx]

**Supporting Information For:**

**The SOS response-induced cell division inhibitor YneA interacts with FtsW to block septal peptidoglycan synthesis in *Bacillus subtilis***

**Authors**

Wenjie Yang^1,2,3^, Chao Wang^3^, Yuanyuan Cui^1, 2^, Wanting Jiang^3^, Shimin Zhu^4^, Changjiang Dong^4^, Joe Lutkenhaus^5^, Xiangdong Chen^3^*, and Shishen Du^1,2^*

**Affiliation**

1 State Key Laboratory of Metabolism and Regulation in Complex Organisms, College of Life Sciences, Wuhan University, Wuhan, Hubei, China

2 Hubei Key Laboratory of Cell Homeostasis, College of Life Sciences, Wuhan University, Wuhan, Hubei, China

3 State Key Laboratory of Virology and Biosafety, College of Life Sciences, Wuhan University, Wuhan, Hubei, China

4 Key Laboratory of Combinatorial Biosynthesis and Drug Discovery, Ministry of education, School of Pharmaceutical Sciences, Wuhan University, Wuhan, China.

5 Department of Microbiology, Molecular Genetics and Immunology, University of Kansas Medical Center, Kansas City, Kansas, USA

*** To whom correspondence should be addressed:**

Shishen Du

State Key Laboratory of Metabolism and Regulation in Complex Organisms,

Wuhan University, Wuhan, Hubei, China

e-mail: [ssdu@whu.edu.cn](mailto:ssdu@whu.edu.cn)

**Supporting Figures and Legends**

**
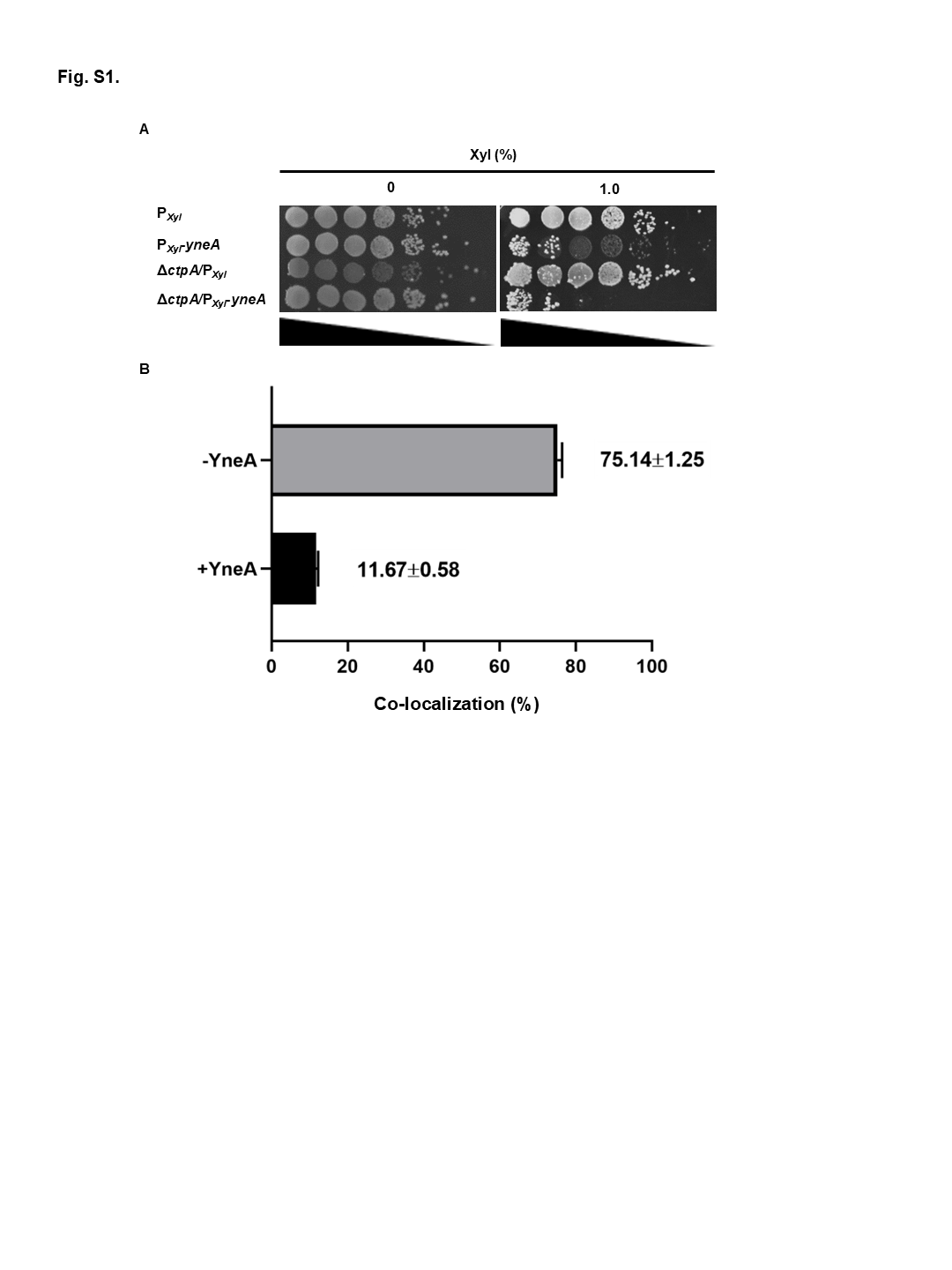
**

**Fig. S1 Toxicity test of YneA.**

*A***,** YneA overexpression blocks cell growth effectively in a *ctpA* deletion strain. Plasmids pYW1 (pHY300-p*_xyl_*) and pYW3 (pYW1-*yneA*) were transformed into strain 168 and strain YW3 (168, *ΔctpA*) on LB plates. Each resulting strain was grown at 37 °C in exponential phase, serially diluted and spotted on LB plates with or without 1.0% Xylose to induce YneA overexpression. Plates were incubated at 37 °C overnight and photographed. *B***,** quantification of the co-localization of HADA bands and EzrA-GFP rings in cells with or without YneA overexpression. Number of HADA bands/EzrA-GFP rings analyzed (No. of cells without YneA overexpression: 598/792; No. of cells overexpressing YneA: 113/964).


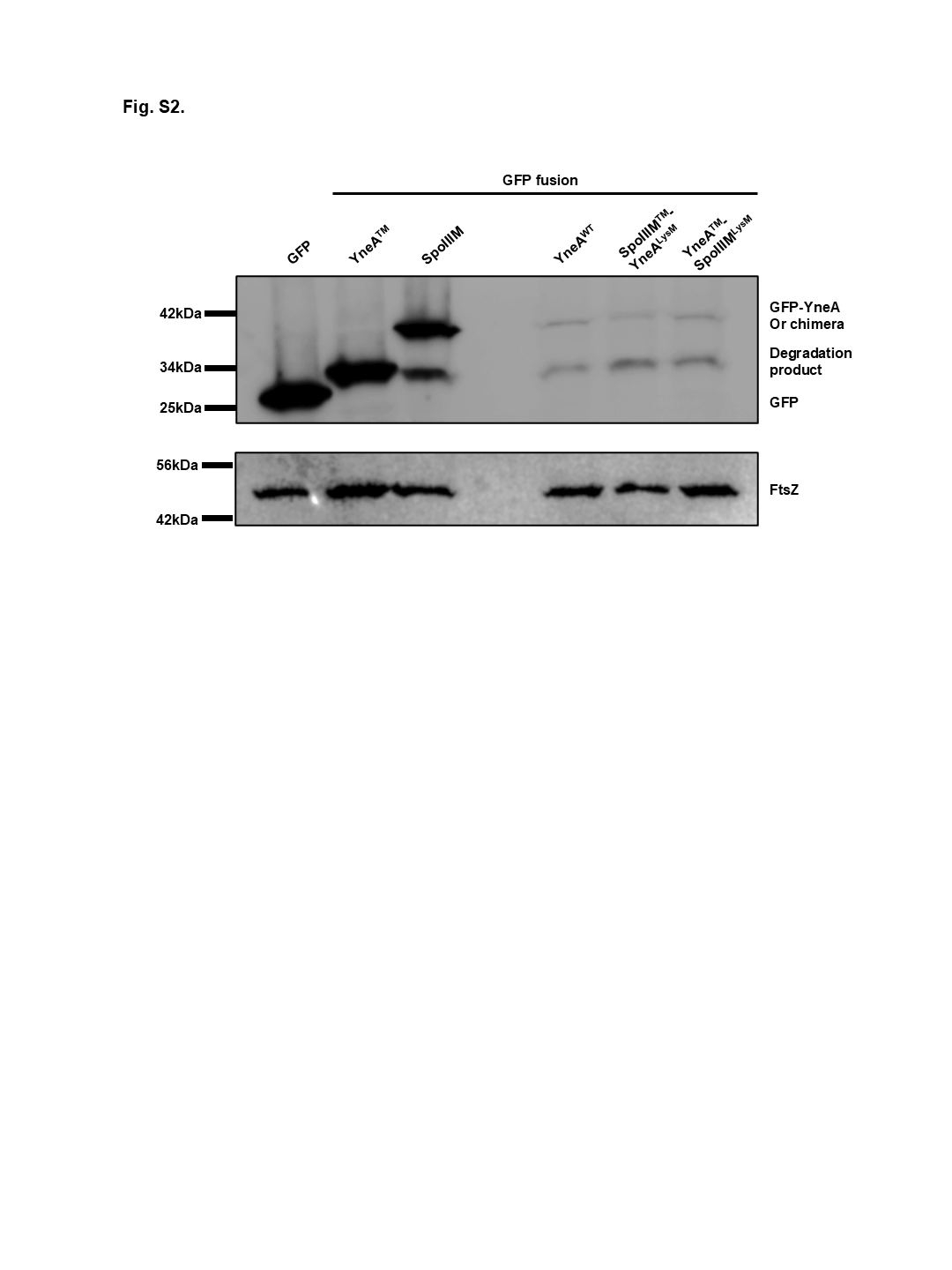


**Fig. S2 Determination of the protein level of GFP fusions of YneA or its chimeras.**

The test was done in strain YW3 (168, *ΔctpA*) carrying plasmid pYW4 (pYW1-*gfp*), pYW29 (pYW1-*gfp*-*spoIIIM*), pYW5 (pYW1-*gfp*-*yneA*) or its derivatives carrying *yneA* mutations. Details about the western blot are described in Experimental procedures. Three biological replicates were carried out, similar results were obtained and only one representative gel image was shown.


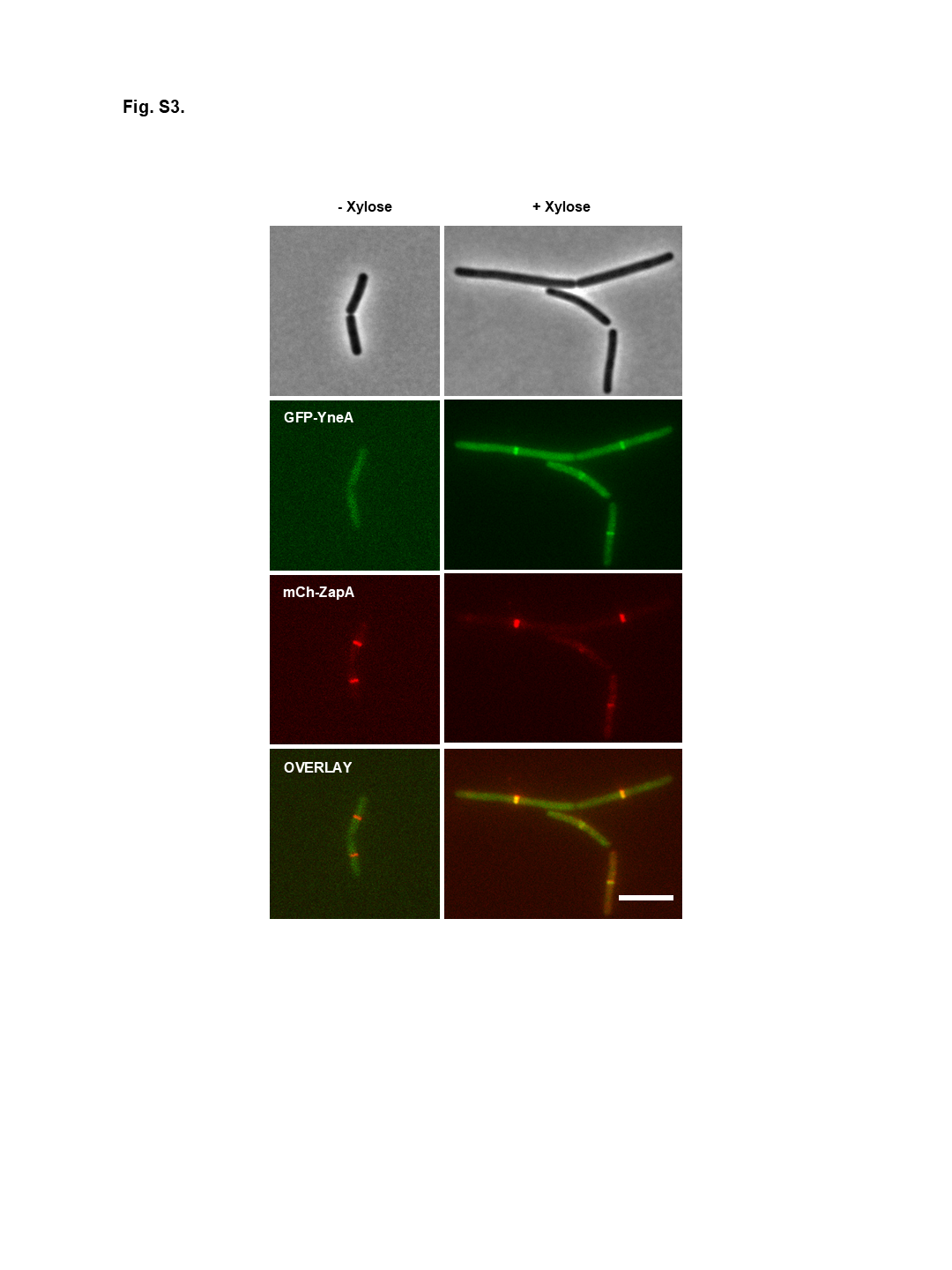


**Fig. S3 GFP-YneA co-localizes with mCherry-ZapA at the division site.**

The localization of GFP-YneA and mCherry-ZapA was assessed in strain YW11 [168, *ΔctpA*/pYW5 (pYW1-*gfp*-*yneA*), pYW10 (pHT01K-*mcherry*-*zapA*)]. The strain was grown at 37 °C in exponential phase in LB medium with 20 µM IPTG to induce the expression of mCherry-ZapA, with or without 0.5% Xylose to induce GFP-YneA expression for 2 hours before imaging. Three biological replicates were carried out, similar results were obtained and only representative images were shown. Scale bar, 5 µm.


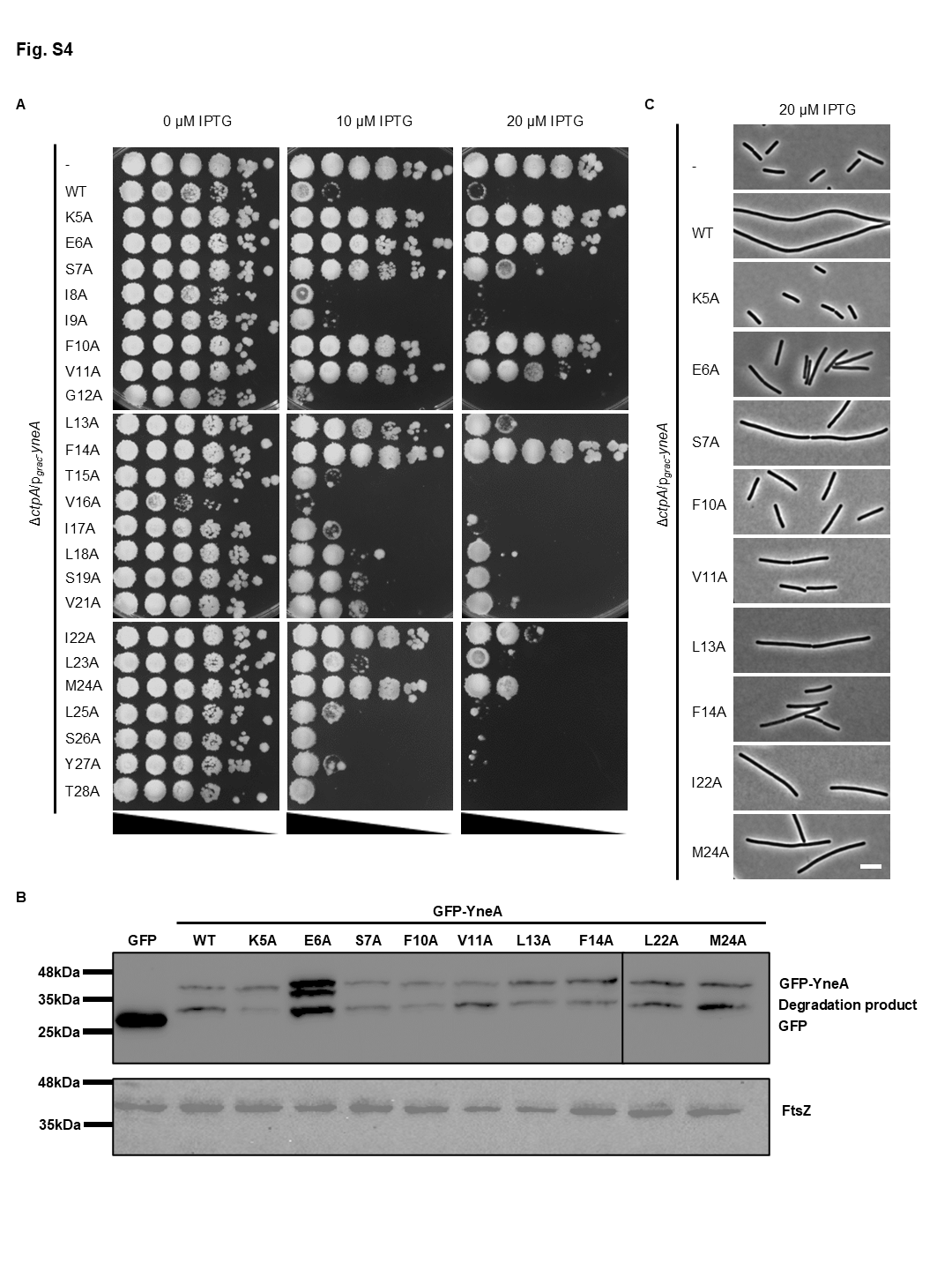


**Fig. S4 Mutations in the transmembrane domain of YneA reduce its cell division inhibitory activity.**

*A***,** spot test of the toxicity of YneA mutants. Plasmid pHT01K, pYW12 (pHT01K-*yneA*) or its derivatives carrying *yneA* mutations were transformed into strain YW3 (168, *ΔctpA*) on LB plates. Each resulting strain was grown at 37 °C in exponential phase, serially diluted and spotted on LB plates with or without 10 or 20 µM IPTG to induce YneA overexpression. Plates were incubated at 37 °C overnight and photographed. *B***,** representative images of cells overexpressing YneA or its variants. Each strain was grown at 37 °C in exponential phase in LB medium with 20 µM IPTG to induce the overexpression of YneA or its mutants for 2 hours. Cells were immobilized on 2% agarose pads for photography. Scale bar, 5 µm. *C***,** western blotting analysis of the protein level of YneA or its variants. The test was done in strain YW3 (168, *ΔctpA*) carrying plasmid pYW4 (pYW1-*gfp*), pYW5 (pYW1-*gfp*-*yneA*) or its derivatives carrying *yneA* mutations. Details about the western blot are described in Experimental procedures. Three biological replicates were carried out for each experiment.


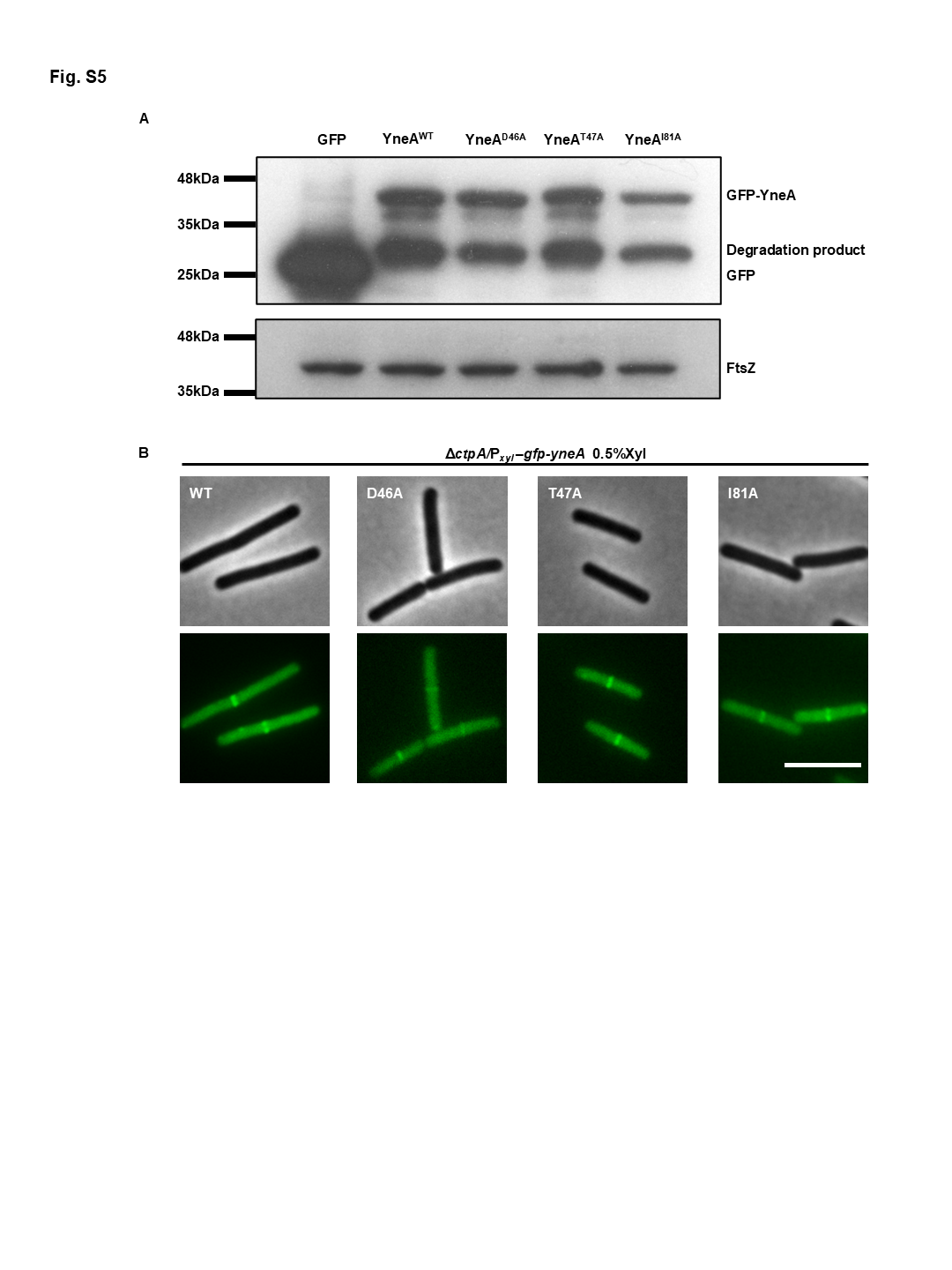


**Fig. S5 Determination of the stability and localization of YneA variants containing mutations in the LysM domain.**

*A*, western blot to test the stability of YneA mutants. The test was done in strain YW3 (168, *ΔctpA*) carrying plasmid pYW4 (pYW1-*gfp*), pYW5 (pYW1-*gfp*-*yneA*) or its derivatives carrying *yneA* mutations. Details about the western blot were described in Experimental procedures. *B***,** representative images of localization of YneA mutants. The localization of YneA or its mutants was tested in strain YW3 (168, *ΔctpA*) carrying plasmid pYW5 (pYW1-*gfp*-*yneA*) or its derivatives containing *yneA* mutations. These resulting strains were grown at 37 °C in exponential phase in LB medium with 0.5% Xylose to induce the expression of GFP fusion proteins for 2 hours. Cells were then immobilized on 2% agarose pads for photography. Three biological replicates were carried out for each test. Scale bar, 5 µm.

**
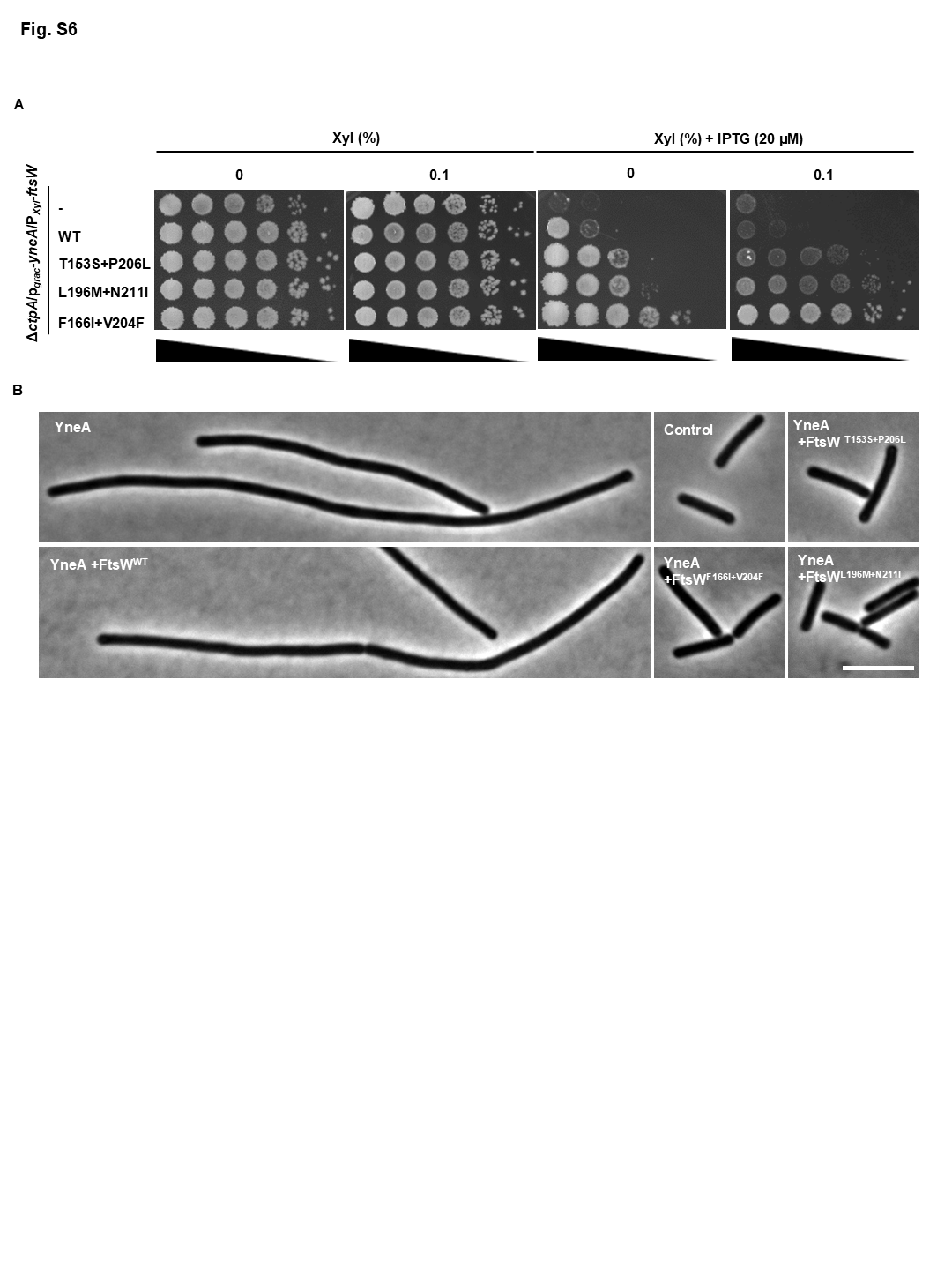
**

**Fig. S6 Screening for FtsW mutations suppressing YneA overexpression.**

*A***,** spot test of the ability of FtsW mutants to suppress the toxicity of YneA overexpression. The test was done in strain YW13 [168, *ΔctpA*/pYW12 (pHT01K*-yneA*)] carrying plasmids pYW1, pYW123 (pYW1-*ftsW*) or its derivatives carrying *ftsW* mutations. The spot test was performed as in Fig 2*B*. *B***,** cell morphology of the strains from (*A*). Each strain in (*A*) was grown at 37 °C in exponential phase in LB medium with 20 µM IPTG to induce the expression of YneA and 0.1% Xylose to induce the expression of FtsW or its mutants for 2 hours. Cells were immobilized on 2% agarose pads for photography. Three biological replicates were carried out for each test. Scale bar, 5 µm.


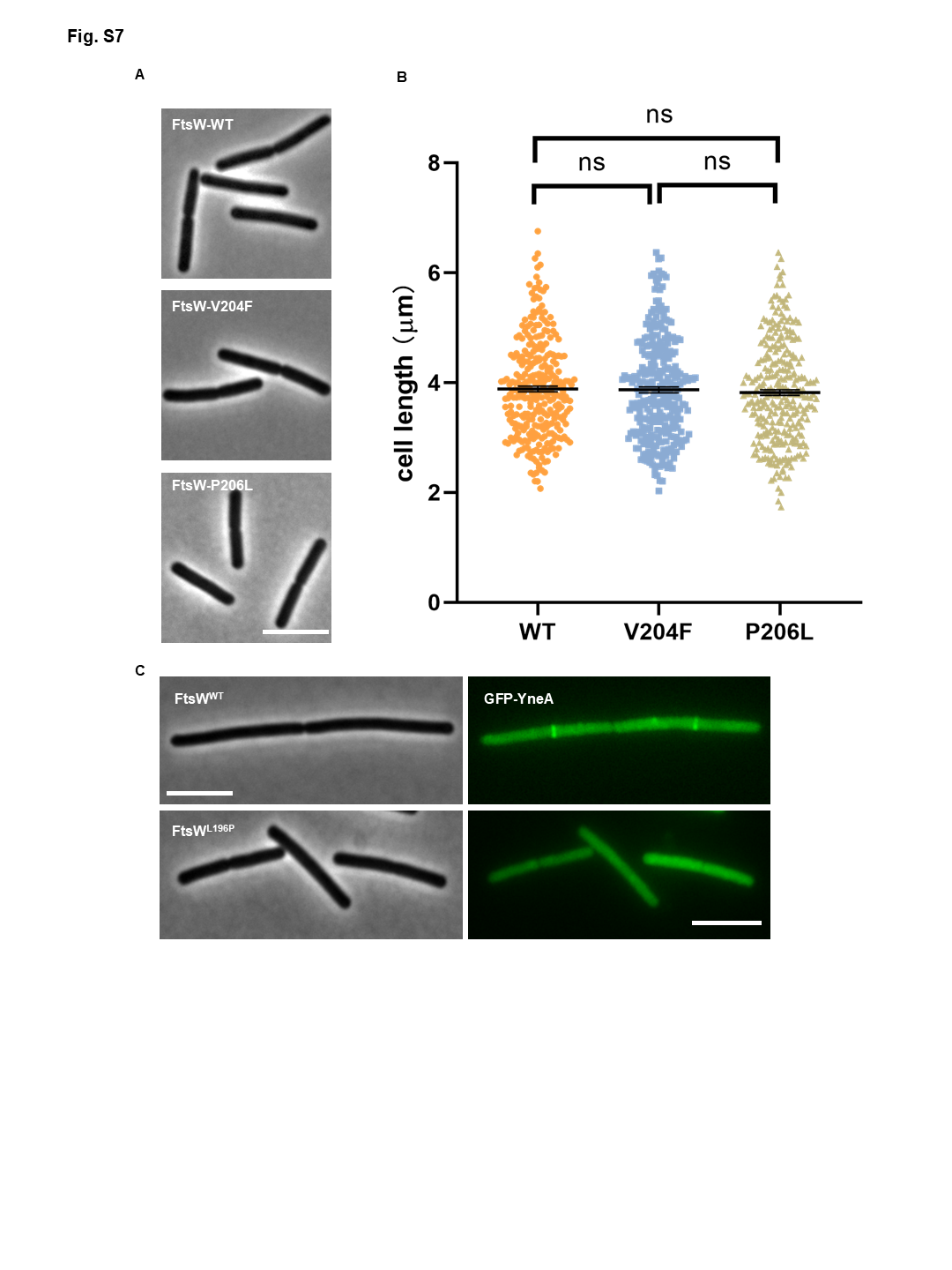


**Fig. S7 YneA resistant FtsW mutations do not result in smaller cells.**

*A***,** morphology of WT, FtsW^V204F^ and FtsW^P206L^ cells. Strains 168, YW147 (168, *ftsW^V204F^*), and YW148 (168, *ftsW^P206L^*) were grown at 37 °C in exponential phase and cells were then immobilized on 2% agarose pads for photography. *B***,** cell length distribution of WT, FtsW^V204F^ and FtsW^P206L^ cells. The number of cells analyzed is 300 for each strain. Data were presented as the mean with standard error of the mean (SEM). ns, not significant (P>0.05), two-tailed t-test. *C***,** representative images of GFP-YneA localization in wild type or FtsW^L196P^ cells. Plasmid pYW13 (pHT01K-*gfp*-*yneA*) was transformed into strains YW3 (168, *ΔctpA*) and YW395 (168, *ΔctpA ftsW^L196P^*). These resulting strains were grown at 37 °C in exponential phase. 10 µM IPTG was added to induce the expression of GFP-YneA for 2 hours before imaging. Three biological replicates were carried out for the test. Scale bars in (*A*) and (*C*), 5 µm.


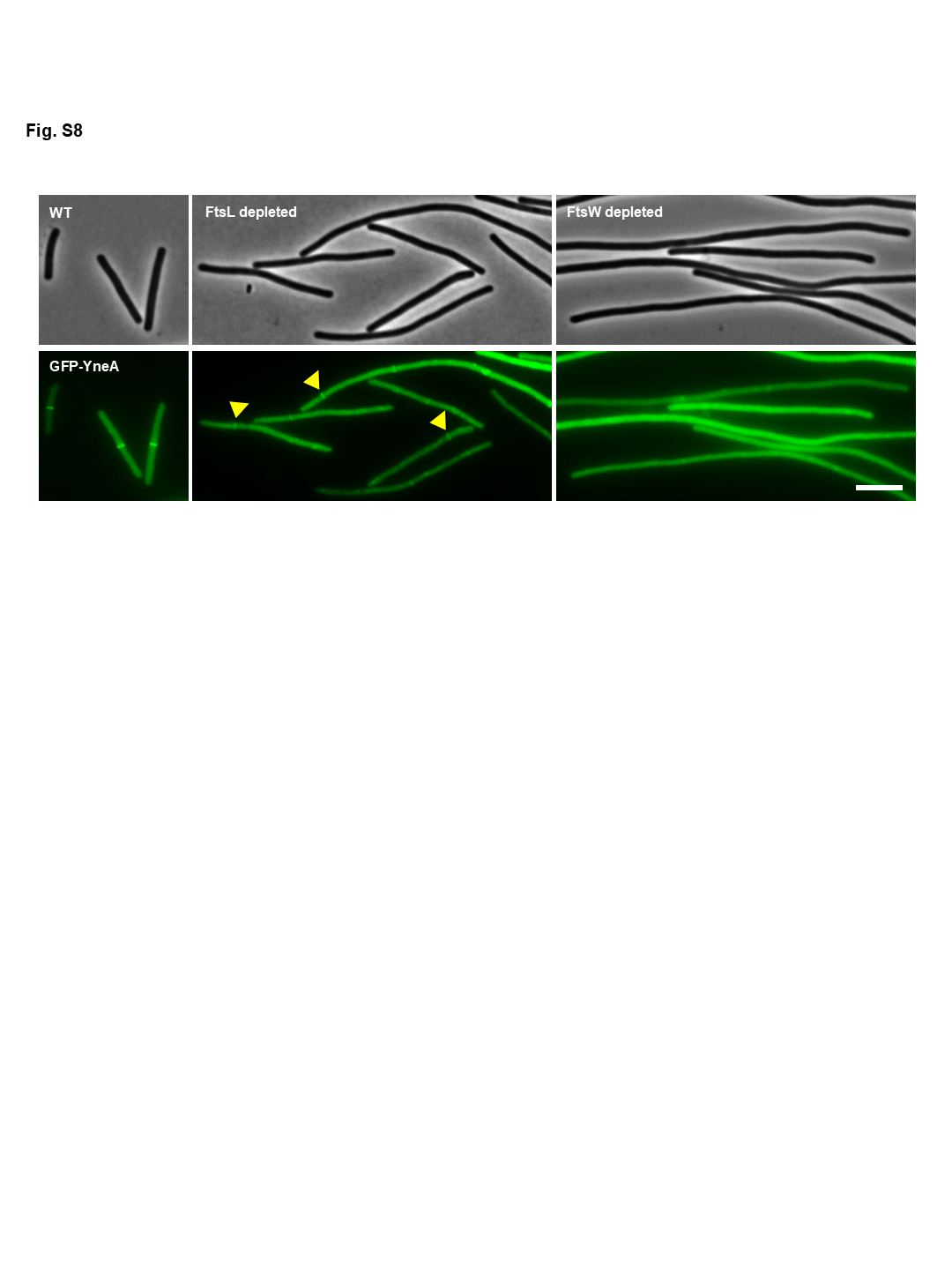


**Fig. S8 Localization of YneA depends on FtsW, but not FtsL.**

Overnight cultures of strain YW3 (168, *ΔctpA*), YW94 (GYQ203, *ftsL_native_::*P_43_ *ΔctpA*) and YW109 (GYQ81, *ΔctpA* *ΔftsW_native_*) carrying plasmid pYW5 (pYW1-*gfp*-*yneA*) were grown at 37 °C in exponential phase in LB medium with 50 µM IPTG (except strain YW3). Cells were collected by centrifugation and washed twice with fresh LB medium to remove the IPTG, followed by resuspension in the same volume of LB medium. These cultures were then diluted 1:25 in fresh LB medium and grown at 37 °C. After the removal of IPTG for 2 hours, 0.5% Xylose was added to the cultures to induce the expression of GFP-YneA for another 2 hours before imaging. Yellow triangles indicate the presumptive division sites marked by GFP-YneA. Scale bar, 5 µm.


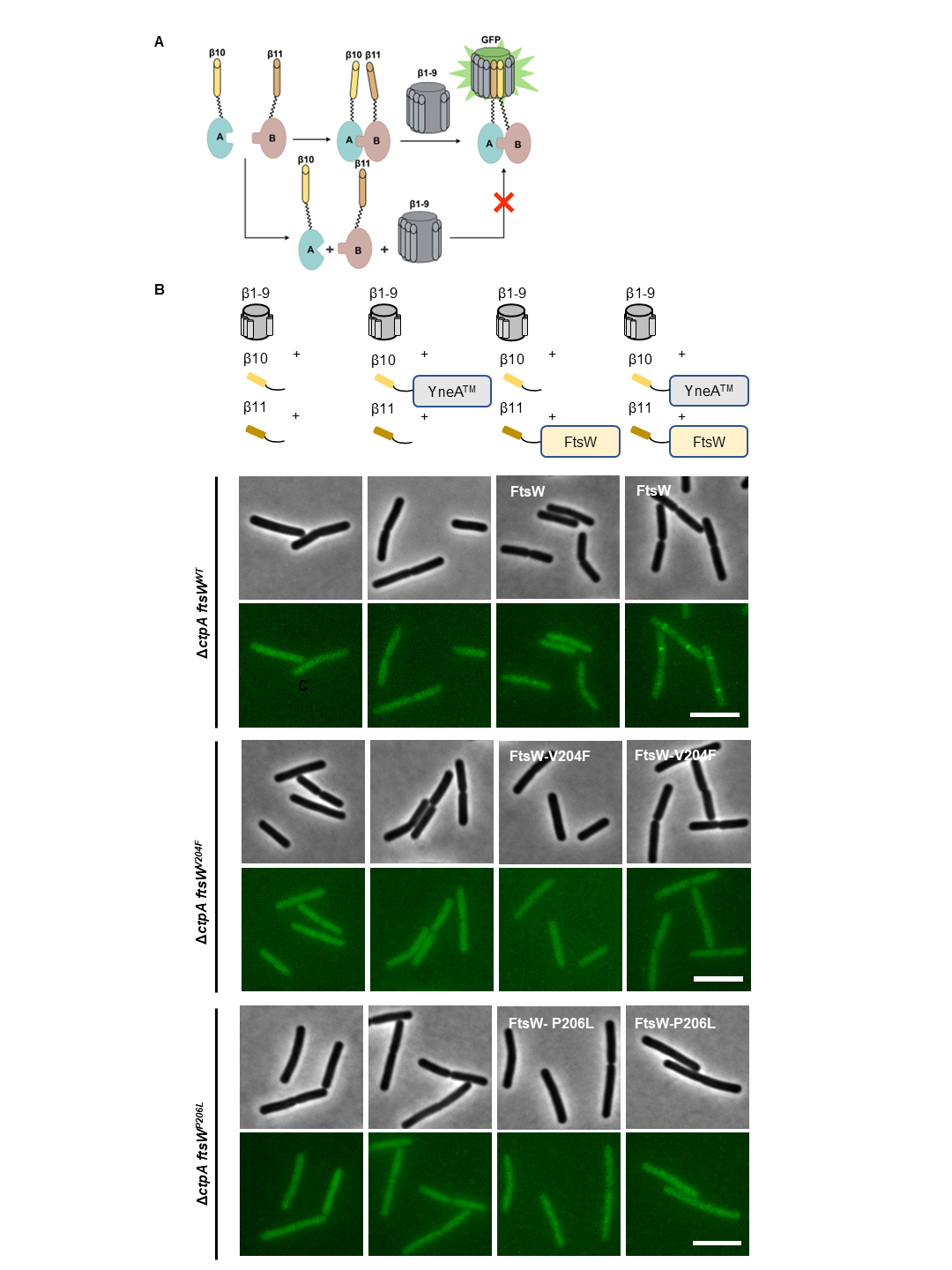


**Fig. S9 YneA^TM^ interacts with FtsW in Split-FP assay.**

*A*, a diagram showing the principle of the Split-FP assay. The super folder GFP is divided into three parts, β1-9, β10 and β11. Two proteins to be tested for interaction are tagged with β10 and β11, respectively. If the two proteins interact with each other, β10 and β11 will be in close proximity and interact with β1-9, leading to the reconstitution of super folder GFP and exhibits fluorescence. *B*, Split-FP assay to test the interaction between YneA^TM^ and FtsW or its mutants. Strains YW3 (168, *ΔctpA*), YW149 (168, *ΔctpA* *ftsW^V204F^*), and YW150 (168, *ΔctpA* *ftsW^P206L^*) carrying split-FP plasmids were grown at 37 °C for 2 hours followed by a shift to 30 °C with 1 mM IPTG for another 3 hours. Cells were immobilized on 2% agarose pads for photography. Scale bar, 5 µm.


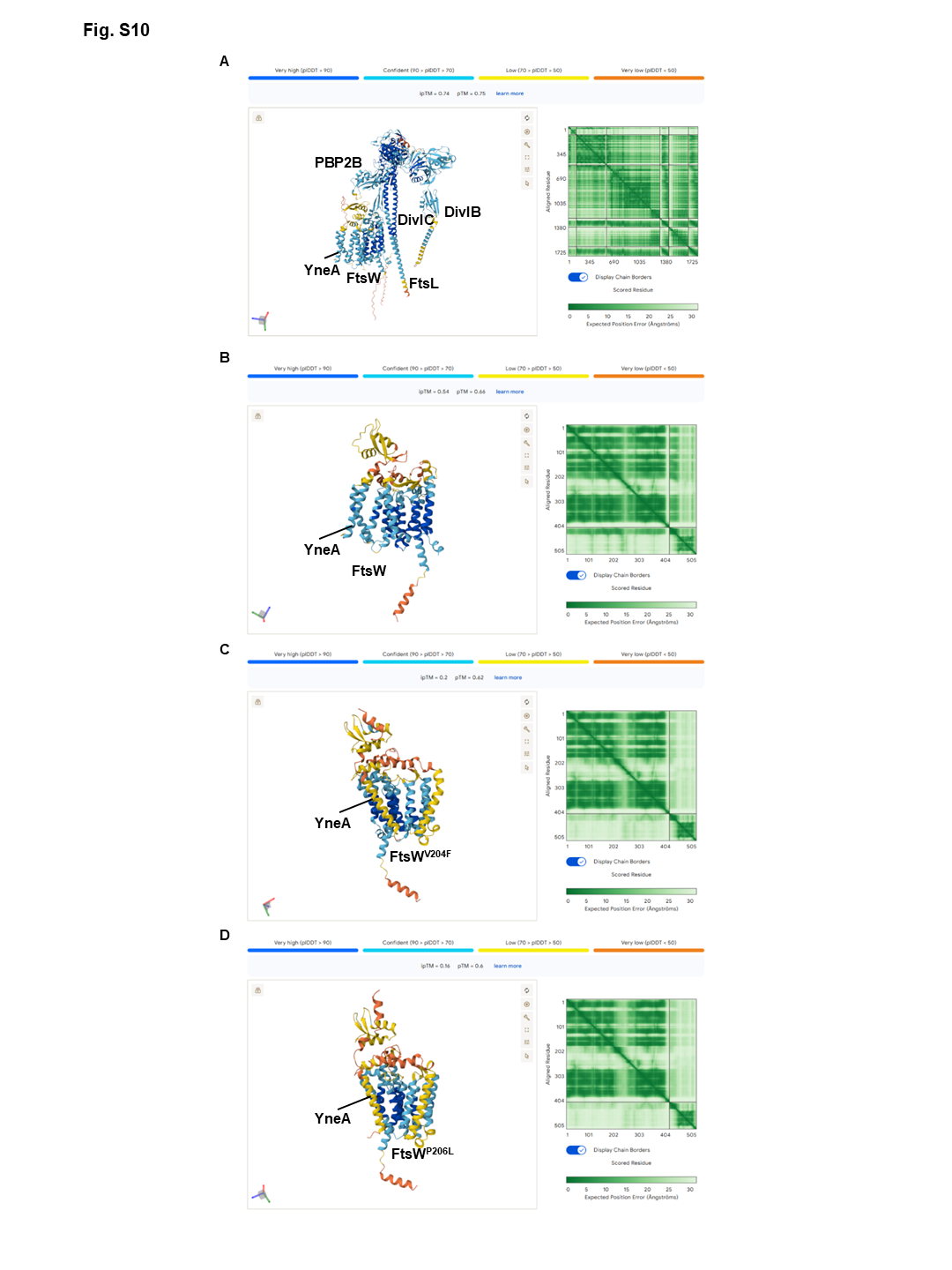


**Fig. S10 Structural models of YneA in complex with sPG synthetic complex, wild type FtsW or its mutants predicted by AlphaFold 3.**

*A***,** a structural model of a multi-protein complex consisting of YneA and the sPG synthetic complex (FtsW-PBP2B-DivIB-FtsL-DivIC; ipTM = 0.74, pTM = 0.75) predicted by AlphaFold 3. (*B*-*D*) structural models of the YneA-FtsW/FtsW^V204F^/FtsW^P206L^ complexes predicted by AlphaFold 3. *B*, YneA-FtsW: ipTM = 0.54, pTM = 0.66; *C***,** YneA- FtsW^V204F^: ipTM = 0.2, pTM = 0.62; *D*, YneA-FtsW^P206L^: ipTM = 0.16, pTM = 0.6. The structures are colored by per-residue pLDDT: *blue* (≥90, very high confidence), *cyan* (70-90, confident), *yellow* (50-70, low confidence), and *orange* (<50, very low confidence).The corresponding Expected Position Error is shown on the right of the predicted structural model, respectively.


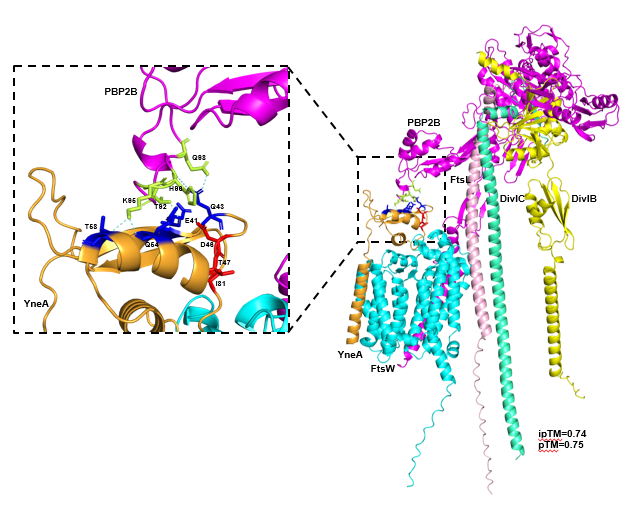


**Fig. S11 Residues important for peptidoglycan binding in the LysM domain of YneA are not in the putative interaction interface between YneA and PBP2B.**

In this structural model of YneA-FtsW-PBP2B-FtsL-DivIC-DivIB (ipTM = 0.74, pTM = 0.75), the LysM domain of YneA appears to interact with the pedestal domain of PBP2B. Contacting residues in PBP2B are colored *light green* and numbered, while those residues in YneA are colored *blue* and numbered. Residues important for PG binding and toxicity of YneA are colored *red* and numbered. Apparently, the residues important for PG binding are not in the putative interaction interface.

**
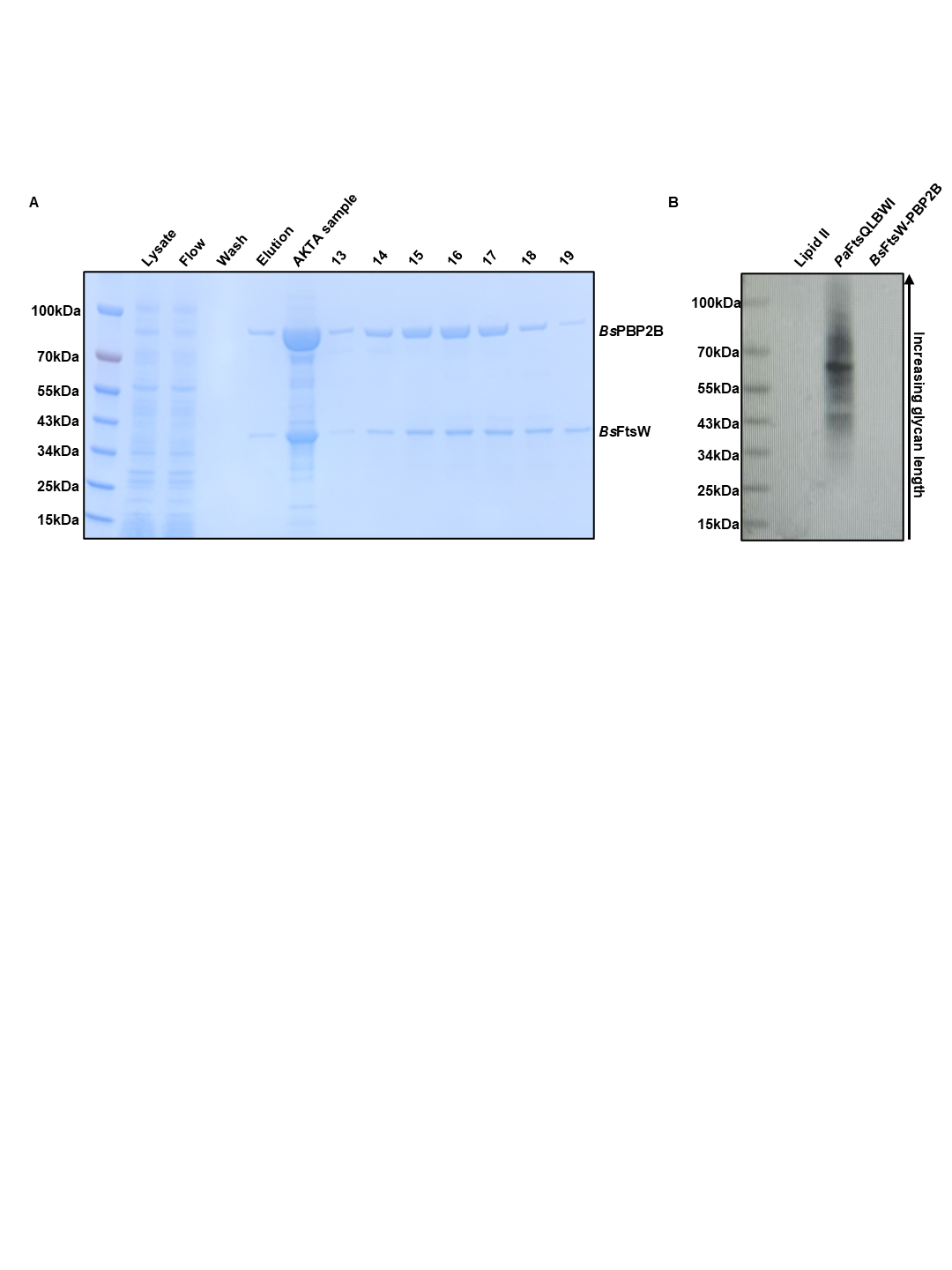
**

**Fig. S12 Purification of the FtsW-PBP2B complex and examination of its peptidoglycan polymerase activity.**

*A***,** purification of the *Bs*FtsW-PBP2B complex. FtsW and PBP2B were co-expressed in *E*. *coli* cells (c43); Flag-tagged FtsW was used as bait to co-purify untagged PBP2B on an Anti-Flag affinity column. The samples were analyzed by SDS-PAGE. *B***,** in vitro analysis of Lipid II polymerization by the *Bs*FtsW-PBP2B complex. Negative control: Lipid II alone; Positive control: Lipid II with *Pa*FtsQLBWI (*Pseudomonas aeruginosa*). Details about the Lipid II polymerization assay are described in Experimental procedures.

**
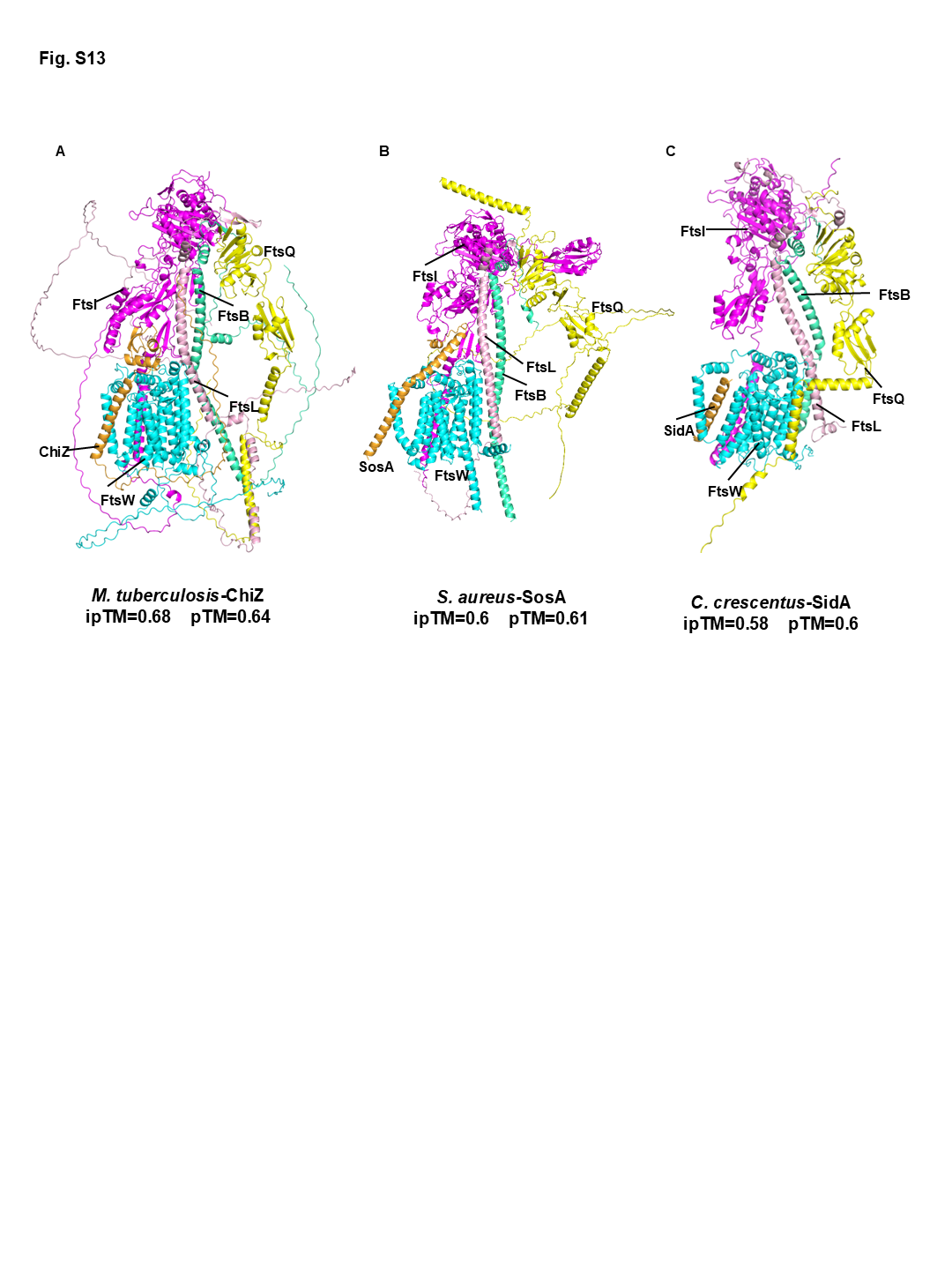
**

**Fig. S13 Structural models of DNA damage induced cell division inhibitors in complex with corresponding sPG synthetic complex predicted by AlphaFold 3.**

An interaction interface is predicted to exist between ChiZ (*M. tuberculosis*)/SosA (*S. aureus*)/SidA (*C. crescentus*) and FtsW. *A***,** a structural model of a multi-protein complex consisting of ChiZ and the sPG synthetic complex in *M. tuberculosis* (ipTM = 0.68, pTM = 0.64). ChiZ, FtsL, FtsB, FtsQ, FtsW and FtsI are colored *bright orange*, *light pink*, *green cyan*, *yellow*, *cyan* and *magenta*, respectively. *B***,** a structural model of a multi-protein complex consisting of SosA and the sPG synthetic complex in *S. aureus* (ipTM = 0.6, pTM = 0.61). SosA, FtsL, FtsB, FtsQ, FtsW and FtsI are colored *bright orange*, *light pink*, *green cyan*, *yellow*, *cyan* and *magenta*, respectively. *C***,** structural models of multi-protein complexes consisting of SidA and the sPG synthetic complex in *C. crescentus* (ipTM = 0.58, pTM = 0.6). SidA, FtsL, FtsB, FtsQ, FtsW and FtsI are colored *bright orange*, *light pink*, *green cyan*, *yellow*, *cyan* and *magenta*, respectively.

**
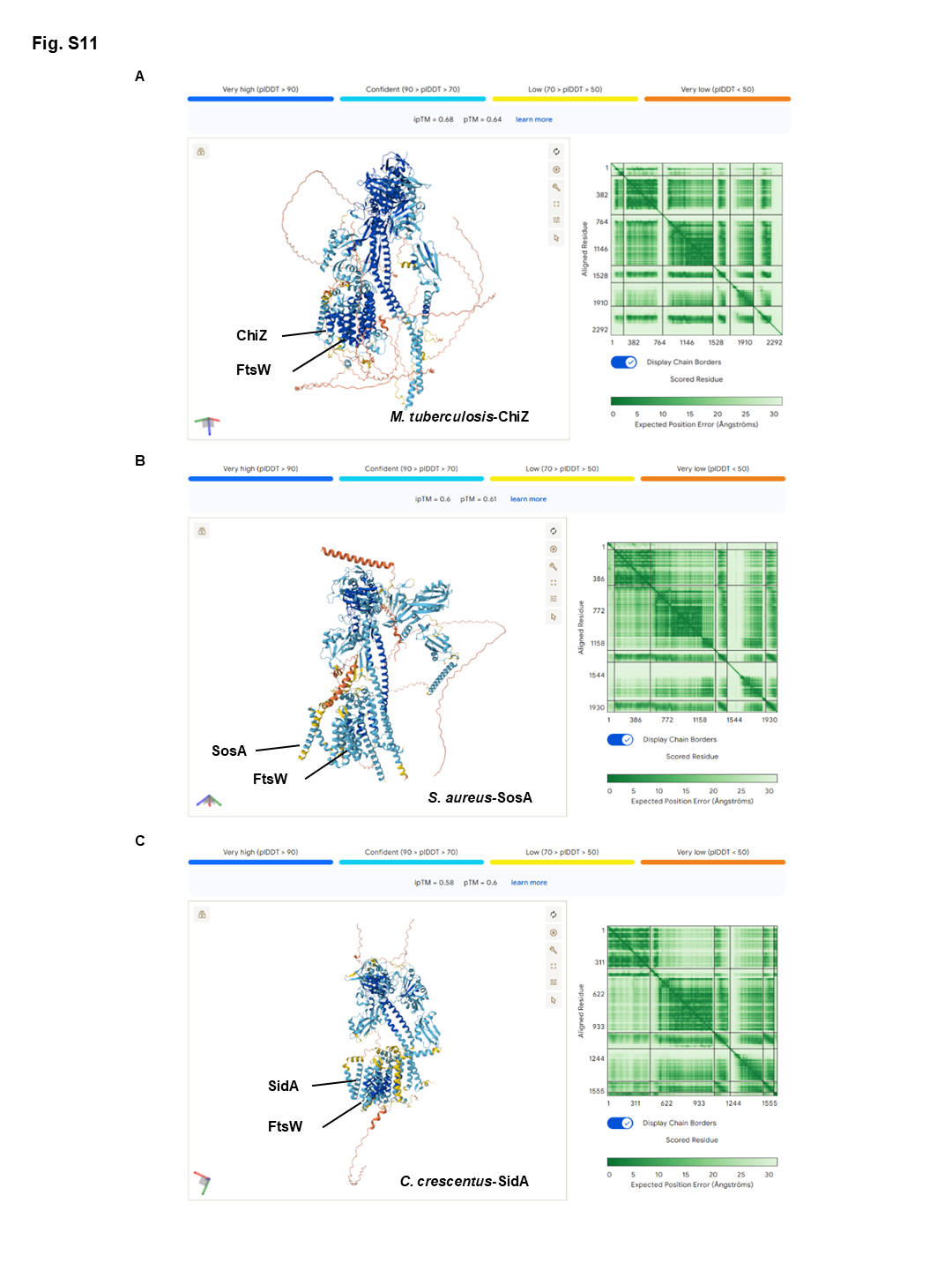
**

**Fig. S14 Structural models of DNA damage induced cell division inhibitors in complex with corresponding sPG synthetic complex predicted by AlphaFold 3.** An interaction interface is predicted to exist between ChiZ (*M. tuberculosis*)/SosA (*S. aureus*)/SidA (*C. crescentus*) and FtsW. *A***,** a structural model of a multi-protein complex consisting of ChiZ and the sPG synthetic complex in *M. tuberculosis* (ipTM = 0.68, pTM = 0.64). *B***,** a structural model of a multi-protein complex consisting of SosA and the sPG synthetic complex in *S. aureus* (ipTM = 0.6, pTM = 0.61). *C***,** structural models of multi-protein complexes consisting of SidA and the sPG synthetic complex in *C. crescentus* (ipTM = 0.58, pTM = 0.6). The structures are colored by per-residue pLDDT: *blue* (≥90, very high confidence), *cyan* (70-90, confident), *yellow* (50-70, low confidence), and *orange* (<50, very low confidence).The corresponding Expected Position Error is shown on the right of the predicted structural model, respectively.


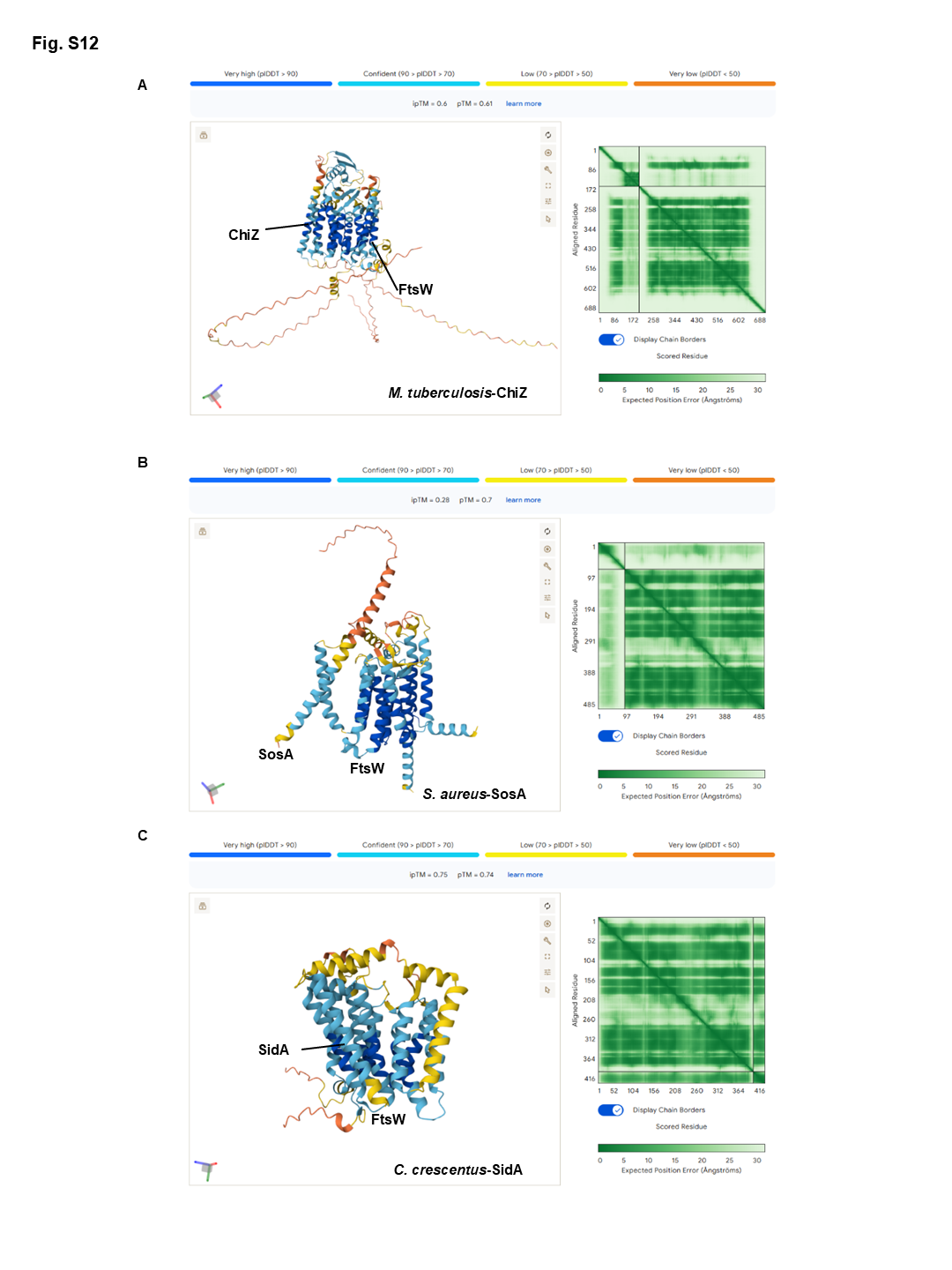


**Fig. S15 Structural models of DNA damage induced cell division inhibitors in complex with FtsW predicted by AlphaFold 3.**

*A***,** a structural model of ChiZ-FtsW complex (ipTM = 0.6, pTM = 0.61) in *M. tuberculosis. B***,** a structural model of SosA-FtsW complex (ipTM = 0.28, pTM = 0.7) in *S. aureus.* *C***,** a structural model of SidA-FtsW complex (ipTM = 0.75, pTM = 0.74) in *C. crescentus.* The structures are colored by per-residue pLDDT: *blue* (≥90, very high confidence), *cyan* (70-90, confident), *yellow* (50-70, low confidence), and *orange* (<50, very low confidence).The corresponding Expected Position Error is shown on the right of the predicted structural model, respectively.

**Table S1. Quantification of the localization of division proteins in Fig. 1*A*.**

| **Protein** | **Septal localization (mean ± SD %)** | **Cell number** |
| --- | --- | --- |
| FtsZ-GFP (-Xyl) | 66.23 ± 6.60 | 347 |
| FtsZ-GFP (+Xyl) | 57.49 ± 11.43 | 284 |
| EzrA-GFP (-Xyl) | 65.17 ± 9.22 | 328 |
| EzrA-GFP (+Xyl) | 58.12 ± 0.63 | 294 |
| GFP-FtsL (-Xyl) | 61.45 ± 7.22 | 351 |
| GFP-FtsL (+Xyl) | 65.56 ± 5.42 | 199 |
| GFP-PBP2B (-Xyl) | 49.26 ± 6.77 | 321 |
| GFP-PBP2B (+Xyl) | 50.66 ± 9.08 | 262 |
| GFP-FtsW (-Xyl) | 58 ± 2.70 | 309 |
| GFP-FtsW (+Xyl) | 58.59 ± 6.77 | 207 |

-Xyl: without YneA overexpression; +Xyl: 1.5% Xylose was added to induce YneA overexpression.

**Table S2. Average cell length of the strains in Fig. 2*C*.**

| **Strain** | **Cell length (mean ± SD μm)** | **Cell number** |
| --- | --- | --- |
| YW3/pHT01K (control) | 3.19 ± 0.17 | 400 |
| YW3/pYW12 (YneA) | 29.42 ± 2.51 | 240 |
| YW3/pYW14 (YneA^TM^) | 3.68 ± 0.21 | 364 |
| YW3/pYW26 (SpoIIIM) | 3.55 ± 0.15 | 350 |
| YW3/pYW27 (SpoIIIM^TM^-YneA^LysM^) | 3.61 ± 0.03 | 375 |
| YW3/pYW28 (YneA^TM^-SpoIIIM^LysM^) | 3.40 ± 0.20 | 328 |

**Table S3. The co-localization of GFP-YneA rings and mCherry-ZapA rings in Fig. S3.**

| **Strain** | **Co-localization (mean ± SD %)** | **Number of GFP-YneA /mCherry-ZapA rings** |
| --- | --- | --- |
| YW3/pYW10, pYW5 | 84.54 ± 5.45 | 179/213 |

**Table S4. Quantification of the localization of GFP fusion of YneA or its variants in Fig. 3*A*.**

| **Protein** | **Septal localization (mean ± SD %)** | **Cell number** |
| --- | --- | --- |
| GFP-YneA | 60.26 ± 5.24 | 316 |
| GFP-YneA^TM^ | 57.1 ± 6.20 | 292 |
| GFP-SpoIIIM | 0 ± 0 | 340 |
| GFP-SpoIIIM^TM^-YneA^LysM^ | 0 ± 0 | 358 |
| GFP-YneA^TM^-SpoIIIM^LysM^ | 58.9 ± 3.55 | 286 |

**Table S5. Quantification of the localization of YneA and its mutants in Fig. 3*B*.**

| **Protein** | **Septal localization (mean ± SD %)** | **Cell number** |
| --- | --- | --- |
| GFP-YneA^WT^ | 59.96 ± 7.47 | 348 |
| GFP-YneA^K5A^ | 31.00 ± 3.00 | 312 |
| GFP-YneA^E6A^ | 0 ± 0 | 325 |
| GFP-YneA^F10A^ | 0 ± 0 | 365 |
| GFP-YneA^V11A^ | 0 ± 0 | 356 |
| GFP-YneA^F14A^ | 0 ± 0 | 327 |

**Table S6. Average cell length of the strains in Fig. 4*D*.**

| **Strain** | **IPTG** | **Cell length (mean ± SD μm)** | **Cell number** |
| --- | --- | --- | --- |
| YW3/pHT01K (vector) | - | 2.85 ± 0.06 | 200 |
| YW3/pHT01K (vector) | + | 2.97 ± 0.10 | 197 |
| YW3/pYW12 (YneA) | - | 3.28 ± 0.15 | 204 |
| YW3/pYW12 (YneA) | + | 27.97 ± 6.40 | 260 |
| YW3/pYW32 (YneA^D46A^) | - | 3.05 ± 0.11 | 237 |
| YW3/pYW32 (YneA^D46A^) | + | 3.16 ± 0.30 | 245 |
| YW3/pYW33 (YneA^T47A^) | - | 3.12 ± 0.29 | 210 |
| YW3/pYW33 (YneA^T47A^) | + | 3.17 ± 0.16 | 195 |
| YW3/pYW36 (YneA^I81A^) | - | 3.09 ± 0.06 | 181 |
| YW3/pYW36 (YneA^I81A^) | + | 3.39 ± 0.09 | 206 |

- IPTG: without YneA overexpression; + IPTG: 20 μM IPTG was added to induce expression of YneA or its mutants.

**Table S7. Quantification of the localization of YneA and its mutants in Fig. S5*B*.**

| **Protein** | **Septal localization (mean ± SD %)** | **Cell number** |
| --- | --- | --- |
| GFP-YneA^WT^ | 62.81 ± 3.72 | 221 |
| GFP-YneA^D46A^ | 60.4 ± 1.54 | 240 |
| GFP-YneA^T47A^ | 54.94 ± 3.95 | 257 |
| GFP-YneA^I81A^ | 63.03 ± 3.58 | 262 |

**Table S8. Average cell length of the strains in Fig. 5*B*.**

| **Strain** | **Cell length (mean ± SD μm)** | **Cell number** |
| --- | --- | --- |
| YW3/pYW12, pYW1 (control) | 22.18 ± 2.19 | 220 |
| YW3/pYW12, pYW123 (FtsW) | 24.82 ± 3.08 | 216 |
| YW3/pYW12, pYW131 (FtsW^V204F^) | 4.23 ± 0.26 | 226 |
| YW3/pYW12, pYW132 (FtsW^P206L^) | 4.63 ± 0.37 | 200 |

**Table S9. Quantification of the co-localization of GFP-YneA rings and mCherry-ZapA rings in Fig. 5*C*.**

| **Strain** | **Co-localization (mean ± SD %)** | **Number of GFP-YneA /mCherry-ZapA rings** |
| --- | --- | --- |
| YW3/pYW10, pYW5(FtsW) | 78.29 ± 1.10 | 168/215 |
| YW149/pYW10, pYW5(FtsW^V204F^) | 0 ± 0 | 0/280 |
| YW150/pYW10, pYW5(FtsW^P206L^) | 0 ± 0 | 0/248 |

**Construction of strains**

**Construction of *ftsL* or *ftsW* depletion strains**

The strain YW60 (168, *aprE::erm* p*_spac_*-*gfp*-*ftsL ftsL_native_::*p_43_) was constructed by replacing *ftsL_native_* with a constitutive promoter p_43_ using homologous recombination as previously described [1]. Temperature-sensitive plasmid pYW96 (pNNB194, *bla kan* P_T7_-*ftsL*-UP+p_43_+*ftsL-*DN), which contains upstream and downstream homologous regions of *ftsL* and a P_43_ promoter, was electro-transformed into strain GYQ203 on LB plates with kanamycin at 30 °C. The correct transformants were verified by colony PCR using primer pair pNNB194K-F and pNNB194K-R and grown in LB medium at 45 °C with kanamycin, and with 50 µM IPTG to induce the *ftsL* allele at the *aprE* locus. pNNB194K derivatives are unable to replicate in *B. subtilis* at 45 °C owing to the temperature-sensitive replicon, so that they integrate into the chromosomal locus of gene to be deleted (single exchange). The correct integrants were verified by colony PCR using primer pair *ftsL*-out-F/pNNB194K-R or *ftsL*-out-R/pNNB194K-F and then they were grown in LB medium without kanamycin with 50 µM IPTG at 30 °C to promote double exchange and plasmid loss. Finally, the correct *ΔftsL* strain was verified by colony PCR using primer pair *ftsL*-out-F/R and sequencing. The strain YW100 (168, *aprE::erm* p*_spac_-gfp-ftsW* *ΔftsW_native_*) was constructed by deleting *ftsW_native_* using plasmid pYW98 (pNNB194, *bla kan* P_T7_-*ftsW*UD) in the same way as strain YW60, except that the primer pair *ftsW*-out-F/R was used instead.

**Construction of *ctpA* deletion strains**

The strains YW3 (168, *ΔctpA*), YW94 (168, *aprE::erm* P*_spac_*-*gfp*-*ftsL ftsL_native_*::P_43_ *ΔctpA*) and YW109 (168, *aprE*::*erm* P*_spac_-gfp-ftsW ΔctpA* *ΔftsW_native_*) were constructed by deleting *ctpA* using plasmid pYW11 (pNNB194, *bla kan* p_T7_-*ctpA*UD) in the same way as strain YW60. Primer pair *ctpA*-out-F/R was used to verify the successful deletion of *ctpA.*

**Introduction of *ftsW* mutations V204F, P206L and L196P into the chromosome**

The strains YW147 (168, *ftsW^V204F^*), YW148 (168, *ftsW^P206LF^*), YW149 (168, *ΔctpA* *ftsW^V204F^*), YW150 (168, *ΔctpA ftsW^P206L^*), YW395 (168, *ΔctpA ftsW^L196P^*) were constructed by replacing wild type *ftsW* allele with *ftsW^V204F^*, *ftsW^P206L^* or *ftsW^L196P^* using plasmid pYW150 (pNNB194, *bla kan* p_T7_-UP*+ftsW^V204F^+*DN), pYW151 (pNNB194, *bla kan* p_T7_-UP*+ftsW^P206L^+*DN) or pYW417 (pNNB194, *bla kan* p_T7_-UP*+ftsW^L196P^+*DN) in the same way as strain YW60. The correct strains were confirmed by sequencing using primer pair *ftsW*-out-F/R.

**Construction of plasmids**

pYW1

The plasmid pYW1 was constructed by ligation of a HindIII and BamHI digested DNA fragment containing p*_xyl_* into plasmid PHY300 digested with the same enzymes. The DNA fragment was amplified from 168 chromosomal DNA using primer pair p*_xyl_*-F and p*_xyl_*-R.

pYW3

The plasmid pYW3 was constructed by ligation of an BamHI and EcoRI digested DNA fragment containing *yneA* into plasmid pYW1 digested with the same enzymes. The DNA fragment was amplified from 168 chromosomal DNA using primer pair PHY-*yneA*-F and PHY-*yneA*-R.

pYW4

The plasmid pYW4 was constructed by ligation of an BamHI and EcoRI digested DNA fragment containing *gfp* into plasmid pYW1 digested with the same enzymes. The DNA fragment was amplified from pDSW209 (pDSW206, *bla* P_206_*::gfp-mcs*) using primer pair PHY-*gfp*-F and PHY-*gfp*-R.

pYW5

The plasmid pYW5 was constructed by ligation of an BamHI and EcoRI digested DNA fragment containing *gfp*-*yneA* into plasmid pYW1 digested with the same enzymes. A DNA fragment containing *gfp* and the downstream homology region was amplified from pDSW209 (pDSW206, *bla* P_206_*::gfp-mcs*) using primer pair PHY-*gfp*-F and UP-*gfp-yneA*-R, and a DNA fragment containing *yneA* and the upstream homology region was amplified from 168 chromosomal DNA using primer pair DN-*gfp*-*yneA*-F and PHY-*yneA*-R. The DNA fragment containing *gfp*-*yneA* was amplified from the above two DNA fragments using primer pair PHY-*gfp*-F and PHY-*yneA*-R

pYW10

The plasmid pYW10 was constructed by ligation of an BamHI and XmaI digested DNA fragment containing *mcherry-zapA* into plasmid pHT01K digested with the same enzymes. A DNA fragment containing *mcherry* and the downstream homology region was amplified from CYA13 (TB28*, zipA-mCherry-spc^R^*) using primer pair PHT- *mcherry*-F and UP-*mcherry-zapA*-R, and a DNA fragment containing *zapA* and the upstream homology region was amplified from 168 chromosomal DNA using primer pair DN-*mcherry*-*zapA*-F and PHT-*zapA*-R. The DNA fragment containing *mcherry-zapA* was amplified from the above two DNA fragments using primer pair PHT-*mcherry*-F and PHT-*zapA*-R.

pYW11

The plasmid pYW11 was constructed by ligation of an KpnI and BamHI digested DNA fragment containing *ctpA*UD into plasmid pNNB194 digested with the same enzymes. A DNA fragment containing *ctpA-*UP and the downstream homology region was amplified from 168 chromosomal DNA using primer pair *ctpA*-UP-F and *ctpA*-UP-R, and a DNA fragment containing *ctpA-*DN and the upstream homology region was amplified from 168 chromosomal DNA using primer pair *ctpA*-DN-F and *ctpA*-DN-R. The DNA fragment containing *ctpA*UD was amplified from the above two DNA fragments using primer pair *ctpA*-UP-F and *ctpA*-DN-R.

pYW12

The plasmid pYW12 was constructed by ligation of an BamHI and XbaI digested DNA fragment containing *yneA* into plasmid pHT01K digested with the same enzymes. The DNA fragment was amplified from 168 chromosomal DNA using primer pair PHT-*yneA*-F and PHT-*yneA*-R.

pYW13

The plasmid pYW13 was constructed by ligation of an BamHI and XbaI digested DNA fragment containing *gfp-yneA* into plasmid pHT01K digested with the same enzymes. The DNA fragment was amplified from pYW5 (pYW1-*gfp*-*yneA*) using primer pair PHT-*gfp*-F and PHT-*yneA*-R.

pYW14

The plasmid pYW14 was constructed by ligation of an BamHI and XbaI digested DNA fragment containing *yneA^TM^* into plasmid pHT01K digested with the same enzymes. The DNA fragment was amplified from 168 chromosomal DNA using primer pair PHT-*yneA*-F and PHT-*yneA^TM^*-R.

pYW15

The plasmid pYW15 was constructed by ligation of an BamHI and XbaI digested DNA fragment containing *gfp-yneA^TM^* into plasmid pHT01K digested with the same enzymes. The DNA fragment was amplified from pYW5 (pYW1-*gfp*-*yneA*) using primer pair PHT-*gfp*-F and PHT-*yneA^TM^*-R.

pYW19

The plasmid pYW19 was constructed by ligation of an BamHI and EcoRI digested DNA fragment containing *gfp*-*yneA^TM^* into plasmid pYW1 digested with the same enzymes. The DNA fragment was amplified from pYW5 (pYW1-*gfp*-*yneA*) using primer pair PHY-*gfp*-F and PHY-*yneA^TM^*-R.

pYW26

The plasmid pYW26 was constructed by ligation of an BamHI and XbaI digested DNA fragment containing *spoIIIM* into plasmid pHT01K digested with the same enzymes. The DNA fragment was amplified from 168 chromosomal DNA using primer pair PHT-*spoIIIM*-F and PHT-*spoIIIM*-R.

pYW27

The plasmid pYW27 was constructed by ligation of an BamHI and XmaI digested DNA fragment containing *spoIIIM^TM^-yneA^LysM^* into plasmid pHT01K digested with the same enzymes. A DNA fragment containing *spoIIIM^TM^* and the downstream homology region was amplified from 168 chromosomal DNA using primer pair PHT-*spoIIIM*-F and PHT-*spoIIIM^TM^*-R, and a DNA fragment containing *yneA^LysM^* and the upstream homology region was amplified from 168 chromosomal DNA using primer pair *yneA^LysM^*-F and PHT-*yneA*-R. The DNA fragment containing *spoIIIM^TM^-yneA^LysM^* was amplified from the above two DNA fragments using primer pair PHT-*spoIIIM*-F and PHT-*yneA*-R.

pYW28

The plasmid pYW28 was constructed by ligation of an BamHI and XmaI digested DNA fragment containing *yneA^TM^-spoIIIM^LysM^* into plasmid pHT01K digested with the same enzymes. A DNA fragment containing *yneA^TM^* and the downstream homology region was amplified from 168 chromosomal DNA using primer pair PHT-*yneA*-F and *yneA^1-35^*-R, a DNA fragment containing *spoIIIM^LysM^* and the upstream and downstream homology regions was amplified from 168 chromosomal DNA using primer pair *spoIIIM^LysM^*-F and *spoIIIM^LysM^*-R, and a DNA fragment containing *yneA^92-105^* and the upstream homology region was amplified from 168 chromosomal DNA using primer pair *yneA^92-105^*-F and PHT-*yneA*-R. The DNA fragment containing *yneA^TM^-spoIIIM^LysM^* was amplified from the above three DNA fragments using the above three primer pairs.

pYW29

The plasmid pYW29 was constructed by ligation of an BamHI and EcoRI digested DNA fragment containing *gfp*-*spoIIIM* into plasmid pYW1 digested with the same enzymes. A DNA fragment containing *gfp* and the downstream homology region was amplified from pYW5 (pYW1-*gfp*-*yneA*) using primer pair PHY-*gfp*-F and UP-*gfp-spoIIIM*-R, and a DNA fragment containing *spoIIIM* and the upstream homology region was amplified from 168 chromosomal DNA using primer pair DN-*gfp*-*spoIIIM*-F and PHY-*spoIIIM*-R. The DNA fragment containing *gfp*-*spoIIIM* was amplified from the above two DNA fragments using primer pair PHY-*gfp*-F and PHY-*spoIIIM*-R.

pYW30

The plasmid pYW30 was constructed by ligation of an BamHI and EcoRI digested DNA fragment containing *gfp-spoIIIM^TM^-yneA^LysM^* into plasmid pYW1 digested with the same enzymes. A DNA fragment containing *gfp* and the downstream homology region was amplified from pYW5 (pYW1-*gfp*-*yneA*) using primer pair PHY-*gfp*-F and UP-*gfp-spoIIIM*-R, and a DNA fragment containing *spoIIIM^TM^-yneA^LysM^* and the upstream homology region was amplified from pYW27 (pYW1-*spoIIIM^TM^-yneA^LysM^*) using primer pair DN-*gfp*-*spoIIIM*-F and PHY-*yneA*-R. The DNA fragment containing *gfp-spoIIIM^TM^-yneA^LysM^* was amplified from the above two DNA fragments using primer pair PHY-*gfp*-F and PHY-*yneA*-R.

pYW31

The plasmid pYW31 was constructed by ligation of an BamHI and EcoRI digested DNA fragment containing *gfp-yneA^TM^-spoIIIM^LysM^* into plasmid pYW1 digested with the same enzymes. A DNA fragment containing *gfp* and the downstream homology region was amplified from pYW5 (pYW1-*gfp*-*yneA*) using primer pair PHY-*gfp*-F and UP-*gfp-yneA*-R, and a DNA fragment containing *yneA^TM^-spoIIIM^LysM^* and the upstream homology region was amplified from pYW28 (pYW1-*yneA^TM^-spoIIIM^LysM^*) using primer pair DN-*gfp*-*yneA*-F and PHY-*yneA*-R. The DNA fragment containing *gfp-yneA^TM^-spoIIIM^LysM^* was amplified from the above two DNA fragments using primer pair PHY-*gfp*-F and PHY-*yneA*-R.

pYW12 derivatives: pYW32-36, 265-287

The plasmids pYW32-36, 265-287 expressing different alleles of *yneA* were constructed by site-directed mutagenesis using corresponding primer pair listed in Multimedia Component 4.

pYW5 derivatives: pYW37-39, 288-296

The plasmids pYW37-39, 288-296 expressing different alleles of *yneA* were constructed by site-directed mutagenesis using corresponding primer pair listed in Multimedia Component 4.

pYW44

The plasmid pYW44 was constructed by ligation of an BsaI and XbaI digested DNA fragment containing *lysM* into plasmid pE-*sumo* digested with BsaI. The DNA fragment was amplified from 168 chromosomal DNA using primer pair pE-*sumo*-*lysM*-F and pE-*sumo*-*lysM*-R.

pYW44 derivatives: pYW45-47

The plasmids pYW45-47 expressing different alleles of *lysM* were constructed by site-directed mutagenesis using primer pairs *yneA*-D46A-F/R, *yneA*-T47A-F/R and *yneA*-I81A-F/R, respectively.

pYW48

The plasmid pYW48 was constructed by ligation of an BamHI and XbaI digested DNA fragment containing *yneA* into plasmid pHT01 digested with the same enzymes. The DNA fragment was amplified from 168 chromosomal DNA using primer pair PHT-*yneA*-F and PHT-*yneA*-R.

pYW49

The plasmid pYW49 was constructed by ligation of an BamHI and EcoRI digested DNA fragment containing *yneA* into plasmid pKT25 digested with the same enzymes. The DNA fragment was amplified from 168 chromosomal DNA using primer pair T25-*yneA*-F and T25-*yneA*-R.

pYW54

The plasmid pYW54 was constructed by ligation of a HindIII and BamHI digested DNA fragment containing *ftsW* into plasmid pUT18 digested with the same enzymes. The DNA fragment was amplified from 168 chromosomal DNA using primer pair *ftsW*-T18-F and *ftsW*-T18-R.

pYW96

The plasmid pYW96 was constructed by ligation of an KpnI and BamHI digested DNA fragment containing *ftsL-*UP*+*p*_43_+ftsL-*DN into plasmid pNNB194 digested with the same enzymes. A DNA fragment containing *ftsL-*UP and the downstream homology region was amplified from 168 chromosomal DNA using primer pair *ftsL-*UP-F and *ftsL-*UP-R, a DNA fragment containing p_43_ and the upstream and downstream homology regions was amplified from 168 chromosomal DNA using primer pair p*_43_*-F and p*_43_*-R, and a DNA fragment containing *ftsL-*DN and the downstream homology region was amplified from 168 chromosomal DNA using primer pair *ftsL-*DN-F and *ftsL-*DN-R. The DNA fragment containing *ftsL-*UP*+*p*_43_+ftsL-*DN was amplified from the above three DNA fragments using the above three primer pairs.

pYW98

The plasmid pYW98 was constructed by ligation of an KpnI and BamHI digested DNA fragment containing *ftsW*UD into plasmid pNNB194 digested with the same enzymes. A DNA fragment containing *ftsW-*UP and the downstream homology region was amplified from 168 chromosomal DNA using primer pair *ftsW*-UP-F and *ftsW*-UP-R, and a DNA fragment containing *ftsW-*DN and the upstream homology region was amplified from 168 chromosomal DNA using primer pair *ftsW*-DN-F and *ftsW*-DN-R. The DNA fragment containing *ftsW*UD was amplified from the above two DNA fragments using primer pair *ftsW*-UP-F and *ftsW*-DN-R.

pYW108

The plasmid pYW108 was constructed by ligation of an BamHI and EcoRI digested DNA fragment containing *gfp*-*ftsW* into plasmid pYW1 digested with the same enzymes. A DNA fragment containing *gfp* and the downstream homology region was amplified from pDSW209 (pDSW206, *bla* P_206_*::gfp-mcs*) using primer pair PHY-*gfp*-F and UP-*gfp-ftsW*-R, and a DNA fragment containing *ftsW* and the upstream homology region was amplified from 168 chromosomal DNA using primer pair DN-*gfp*-*ftsW*-F and PHY-*ftsW*-R. The DNA fragment containing *gfp*-*ftsW* was amplified from the above two DNA fragments using primer pair PHY-*gfp*-F and PHY-*ftsW*-R.

pYW114

The plasmid pYW114 was constructed by ligation of an BamHI and EcoRI digested DNA fragment containing *ftsL* into plasmid pYW1 digested with the same enzymes. The DNA fragment was amplified from 168 chromosomal DNA using primer pair PHY-*ftsL*-F and PHY-*ftsL*-R.

pYW122

The plasmid pYW122 was constructed by ligation of an BamHI and EcoRI digested DNA fragment containing *divIC* into plasmid pYW1 digested with the same enzymes. The DNA fragment was amplified from 168 chromosomal DNA using primer pair PHY-*divIC*-F and PHY-*divIC*-R.

pYW123

The plasmid pYW123 was constructed by ligation of an BamHI and EcoRI digested DNA fragment containing *ftsW* into plasmid pYW1 digested with the same enzymes. The DNA fragment was amplified from 168 chromosomal DNA using primer pair PHY-*ftsW*-F and PHY-*ftsW*-R.

pYW124

The plasmid pYW124 was constructed by ligation of an BamHI and KpnI digested DNA fragment containing *pbp2B* into plasmid pYW1 digested with the same enzymes. The DNA fragment was amplified from 168 chromosomal DNA using primer pair PHY-*pbp2B*-F and PHY-*pbp2B*-R.

pYW123 derivatives: pYW125-133

The plasmids pYW125-133 expressing different alleles of *ftsW* were constructed by site-directed mutagenesis using corresponding primer pair listed in S3 Table.

pYW145

The plasmid pYW145 was constructed by ligation of an BamHI and EcoRI digested DNA fragment containing *yneA^TM^* into plasmid pKT25 digested with the same enzymes. The DNA fragment was amplified from 168 chromosomal DNA using primer pair T25-*yneA*-F and T25-*yneA^TM^*-R.

pYW54 derivatives: pYW146, 147

The plasmids pYW146, 147 expressing different alleles of *ftsW* were constructed by site-directed mutagenesis using primer pairs *ftsW*-V204F-F/R and *ftsW*-P206L-F/R.

pYW149

The plasmid pYW149 was constructed by ligation of an KpnI and BamHI digested DNA fragment containing UP*+ftsW+*DN into plasmid pNNB194 digested with the same enzymes. The DNA fragment was amplified from t168 chromosomal DNA using primer pair *ftsW*-UP-F and *ftsW*-DN-R.

pYW149 derivatives: pYW150, 151, 417

The plasmids pYW150, 151, 417 expressing different alleles of *ftsW* were constructed by site-directed mutagenesis using primer pairs *ftsW*-V204F-F/R, *ftsW*-P206L-F/R and *ftsW*-L196P-F/R, respectively.

pYW418

The plasmid pYW418 was constructed by ligation of an BamHI and XbaI digested DNA fragment containing *sfGFP1-9* into plasmid pHT01K digested with the same enzymes. The DNA fragment containing *sfGFP1-9* was amplified from pZWC056 using primer pair *sfGFP1-9*-F/R.

pYW419

The plasmid pYW419 was constructed by ligation of an KpnI and XbaI digested DNA fragment containing *sfGFP10+sfGFP11* into plasmid pYW418 digested with the same enzymes. A DNA fragment containing *sfGFP10* and the downstream homology region was amplified from plasmid pZWC056 using primer pair *sfGFP10-*F/R, and a DNA fragment containing *sfGFP11* and the upstream homology region was amplified from plasmid pZWC056 using primer pair *sfGFP11*-F/R. The DNA fragment containing *sfGFP10+sfGFP11* was amplified from the above two DNA fragments using primer pair *sfGFP10-*F and *sfGFP11*-R.

pYW420

The plasmid pYW420 was constructed by ligation of an KpnI and XbaI digested DNA fragment containing *sfGFP10-yneA^TM^+sfGFP11* into plasmid pYW418 digested with the same enzymes. A DNA fragment containing *sfGFP10* and the downstream homology region was amplified from plasmid pYW419 using primer pair *sfGFP10-*F and UP-*sfGFP10-yneA^TM^-*R, a DNA fragment containing *yneA^TM^* and the homology regions was amplified from 168 chromosomal DNA using primer pair DN-*sfGFP10-yneA^TM^-*F and UP-*yneA^TM^-sfGFP11*-R, and a DNA fragment containing *sfGFP11* and the upstream homology region was amplified from plasmid pYW419 using primer pair DN-*yneA^TM^-sfGFP11*-F and *sfGFP11-*R. The DNA fragment containing *sfGFP10-yneA^TM^+sfGFP11* was amplified from the above three DNA fragments using the above six primers.

pYW421

The plasmid pYW421 was constructed by ligation of an KpnI and XbaI digested DNA fragment containing *sfGFP10+sfGFP11-ftsW^WT^* into plasmid pYW418 digested with the same enzymes. A DNA fragment containing *sfGFP10+sfGFP11* and the downstream homology region was amplified from plasmid pYW419 using primer pair *sfGFP10-*F and UP-*sfGFP11-ftsW^WT^-*R, and a DNA fragment containing *ftsW^WT^* and the upstream homology region was amplified from 168 chromosomal DNA using primer pair DN-*sfGFP11-ftsW^WT^-*F/R. The DNA fragment containing *sfGFP10+sfGFP11-ftsW^WT^* was amplified from the above two DNA fragments using primer pair *sfGFP10-*F and DN-*sfGFP11-ftsW^WT^-*R.

pYW424

The plasmid pYW424 was constructed by ligation of an KpnI and XbaI digested DNA fragment containing *sfGFP10-yneA^TM^+sfGFP11-ftsW^WT^* into plasmid pYW418 digested with the same enzymes. A DNA fragment containing *sfGFP10-yneA^TM^* and the downstream homology region was amplified from plasmid pYW420 using primer pair *sfGFP10-*F and UP-*yneA^TM^-sfGFP11*-R, and a DNA fragment containing *sfGFP11-ftsW^WT^* and the upstream homology region was amplified from plasmid pYW421 using primer pair DN-*yneA^TM^-sfGFP11*-F and DN-*sfGFP11-ftsW^WT^-*R. The DNA fragment containing *sfGFP10-yneA^TM^+sfGFP11-ftsW^WT^* was amplified from the above two DNA fragments using primer pair *sfGFP10-*F and DN-*sfGFP11-ftsW^WT^-*R.

pYW421 derivatives: pYW422, 423

The plasmids pYW422, 423 expressing alleles of *ftsW* were constructed by site-directed mutagenesis using primer pairs *ftsW*-V204F-F/R and *ftsW*-P206L-F/R.

pYW424 derivatives: pYW425, 426

The plasmids pYW425, 426 expressing alleles of *ftsW* were constructed by site-directed mutagenesis using primer pairs *ftsW*-V204F-F/R and *ftsW*-P206L-F/R.

pYW427

The plasmid pYW427 was constructed by ligation of an BamHI and XbaI digested DNA fragment containing *flag-ftsW* into plasmid pHT01K digested with the same enzymes. The DNA fragment was amplified from 168 chromosomal DNA using primer pair *flag*-*ftsW*-F and *flag*-*ftsW*-R.

pYW427 derivatives: pYW428, 429

The plasmids pYW428, 429 expressing alleles of *ftsW* were constructed by site-directed mutagenesis using primer pairs *ftsW*-V204F-F/R and *ftsW*-P206L-F/R.

**References**

1. Connelly MB, Young GM, Sloma A. Extracellular proteolytic activity plays a central role in swarming motility in Bacillus subtilis. J Bacteriol. 2004; 186(13):4159-67. doi: 10.1128/jb.186.13.4159-4167.2004.
